# Multidirectional motion coupling based extreme motion control of distributed drive autonomous vehicle

**DOI:** 10.1038/s41598-022-17351-4

**Published:** 2022-08-01

**Authors:** Kai Wang, Mingliang Yang, Yang Li, Zibin Liu, Haiying Wang, Weiping Ding

**Affiliations:** 1 School of Mechanical Engineering, Southwest Jiaotong University, Chengdu, 610031 Sichuan China; 2 SGS-CSTC Standards Technical Services (Tianjin) Co., Ltd., Tianjin, 300457 China

**Keywords:** Electrical and electronic engineering, Mechanical engineering

## Abstract

To improve the multidirectional motion control accuracy and driving stability of Distributed Drive Autonomous Vehicles (DDAVs) under extreme conditions, the extreme speed estimation method based on dynamic boundary and the multidirectional motion coupling control law design method based on multi degrees of freedom vehicle dynamic model are proposed. The extreme speed estimation method identifies the stable state of DDAVs by the dynamic boundary composed of yaw rate, sideslip angle and roll angle, and then estimates the extreme speed of the vehicle. The design method of multidirectional motion coupling control law adopts eight-degrees-of-freedom (8-DOF) vehicle dynamic model to design path tracking control law, speed tracking control law, yaw stability control law, and active suspension control law at the same time, so as to realize multidirectional motion coupling control. Based on the above method, the Multidirectional Motion Coupling Control System (MMCCS) of DDAVs is designed. The effectiveness of the proposed method is verified by double-line-shifting and serpentine driving simulations under different road adhesion conditions. The superiority of the method is proved by comparing the existing integrated control method.

## Introduction

With the rapid development of automobile technology, the requirements for comfort, safety, efficiency, and mobility of automobiles are increasing^1^. Autonomous vehicles have unique advantages in meeting the needs of users and set off a worldwide research boom. At the same time, distributed drive technology, active suspension technology, and other automotive electric technology are also in deep development. The future of autonomous vehicles will be the comprehensive embodiment of advanced electric and intelligent technology. To facilitate the description, the autonomous vehicle equipped with an active suspension and distributed drive system is called Distributed Drive Autonomous Vehicles (DDAVs) in this paper. DDAVs can realize independent wheel torque control^2^, active suspension control^3^, and trajectory tracking control^4^, which can improve the stability of the vehicle while ensuring the accuracy of trajectory tracking. Because DDAVs are a representative product of automotive electric technology and intelligent technology, people expect them not only to reach the level of the driver but also beyond the level of the driver. DDAVs should have higher transport efficiency and be able to drive at higher speeds under complex conditions. When the vehicles are driving under complex conditions and the speed reaches the extreme of stable driving, the working condition is the extreme condition, which reaches the maximum transportation efficiency. It is of great significance to study the multidirectional motion stability control technology of DDAVs under extreme conditions.

As one of the key technologies to realize autonomous driving, trajectory tracking control mainly includes Speed Tracking Control (STC) and Path Following Control (PFC). The control accuracy of STC and the smoothness of the executive action are the key indicators^5^ to evaluate the control performance. PID control^6^ is the most widely used method in the design of the STC system, and its biggest advantage is that it does not depend on the accurate vehicle longitudinal system model. Besides, the model prediction algorithm^7^, sliding mode algorithm^8^, and fuzzy algorithm^9^ have also been widely used. The challenge of vehicle speed tracking control is how to consider the influence of tire force constraints on vehicle speed under different road conditions and extreme conditions. On this basis, a robust control strategy is designed to improve control accuracy and vehicle motion stability. PFC studies how to control the vehicle steering system to make the vehicle move along the desired path while ensuring the stability and comfort of the vehicle. Pure Pursuit^10^ and Stanley's^11^ methods are the classical path tracking control algorithms proposed earlier. These two algorithms are based on vehicle kinematics and have the characteristics of simplicity and good real-time performance. However, they do not consider the dynamic performance of the vehicle, and generally have a good control effect at low speed and some simple conditions. To adapt to the complex and changeable driving conditions of autonomous vehicles, considering the nonlinear dynamic characteristics, the path tracking control method based on dynamics has been widely studied^12,13^. The path tracking control method based on dynamics often takes the traditional linear two-degrees-of-freedom (2-DOF) model as the reference model^12,13^, and the control algorithms are PID algorithm^14^, model prediction algorithm^15^, sliding mode algorithm^16^, reinforcement learning control^17^.

Distributed drive vehicles can realize Yaw Stability Control (YSC) without affecting the longitudinal motion by wheel torque independent control. There are many studies on the yaw stability control of distributed drive vehicles. The yaw rate and sideslip angle of the traditional linear 2-DOF vehicle dynamic model under steady-state steering is usually used as control reference objectives. The purpose of improving the yaw stability of the vehicle is to reduce the yaw angular velocity or sideslip angle under extreme conditions^18–22^. The commonly used algorithms in YSC research include PID control^18^, fuzzy control^19^, adaptive control^20^, sliding mode control^21^, and optimization control^22^.

The Active Suspension Control (ASC) system can adjust body posture and improve body stability by changing suspension actuation force. At present, there are many studies on vehicle active suspension control^3,23–25^. Usually, the half vehicle model or seven-degrees-of-freedom (7-DOF) vehicle dynamic model considering roll degree of freedom is used as control reference model. In the existing studies, to prevent vehicle rollover due to excessive roll angle, the vehicle roll angle near rollover is taken as the control limit. The commonly used control algorithms in ASC research are sliding mode control^3^, Linear Parameter Varying (LPV) feedforward control^23^, Model Predictive Control (MPC) control^24^, and optimization control^25^.

The trajectory tracking control method, yaw stability control method, or active suspension control method mentioned above can improve the corresponding performance of the vehicle when used alone, and improve the comprehensive dynamic performance of the vehicle^12,26,27^ when combined. In reference^26^, the active front-wheel steering control system and the direct yaw moment control system are designed, and these two systems are introduced into the multi-agent system framework as agents. The Pareto optimal theory is used to realize the integration and optimization control of the two control systems, and the auxiliary steering and the improvement of the yaw stability of the vehicle are achieved. In references^12^, a hierarchical control scheme was proposed to coordinate the working range of PFC and YSC according to the current working condition of the tire, which can improve the trajectory tracking ability and lateral stability of DDAVs. This method considers the coupling characteristics of DDAVs lateral motion and yaw motion and realizes the collaborative control of PFC and YSC through tire force observation. In reference^27^ a collaborative control method of roll stability and yaw stability based on independent control of suspension damping force and wheel torque was proposed. This paper focused on the yaw moment change caused by the change of suspension damping force on axle load. This method considers the coupling characteristics of vehicle roll motion and yaw motion, and realizes the collaborative control of PFC and YSC by changing the yaw moment.

Most of the existing researches use vehicle models with different degrees of freedom to design motion control strategies in various directions, and then stack them to realize multidirectional motion integrated control. Or through the study of the motion coupling mechanism of different degrees of freedom, the compensation method is obtained to realize multidirectional motion cooperative control, to improve the comprehensive dynamic performance of DDAVs. The above research method is difficult to fully consider the control difference caused by multidirectional motion coupling, and it is difficult to give full play to the maximum advantage of DDAVs multi degrees of freedom control. To solve the problem of DDAVs multidirectional motion coupling control, it is necessary to establish the multidirectional motion coupling vehicle dynamic model to design the multidirectional motion control system directly from the coupling essence of DDAVs’ multidirectional dynamic performance. The existing research on DDAVs driving stability control mostly adopts the method of vehicle unstable response trigger control, when the vehicle has an unstable response, the vehicle stability is improved by reducing the yaw rate, the sideslip angle, or the roll angle. This method of stability control is limited when DDAVs are already unstable. Research on vehicle state planning and control based on DDAVs advanced high-precision map and multi-degree-of-freedom controllable advantages remain to be strengthened. At present, there are many algorithms for integrated control of STC, PFC, YSC, and ASC. Among them, sliding mode control has the advantages of fast response, insensitive to parameter changes, no need for system online identification, and simple physical implementation^28^, which is suitable for vehicle dynamic performance control and has been deeply studied by many scholars. In this paper, the sliding mode algorithm is used as the basic algorithm to realize the proposed multidirectional motion coupling control law design method.

In view of the shortcomings of the existing research mentioned above, this paper proposes the extreme speed estimation method based on dynamic boundary and the multidirectional motion coupling control law design method based on multi degrees of freedom vehicle dynamic model and designs the multidirectional motion coupling control system under the extreme condition of DDAVs.

The main contributions are as follows:

1. Based on the dynamic boundary and the optimization design theory, the extreme speed estimation method is proposed to identify the extreme speed of DDAVs stable driving under extreme conditions. This method is suitable for different road adhesion characteristics and different road curvatures. Firstly, the dynamic boundary^29^ is improved, and the composition elements of the dynamic boundary are expanded to yaw rate, sideslip angle, and roll angle. Then, the dynamic boundary is used to construct the extreme speed estimation objective function, and the optimization algorithm is used to solve the extreme speed. Finally, DDAVs are controlled below the extreme speed by STC to avoid tire force exceeding the adhesion limit.
2. To realize the multidirectional motion coupling control of DDAVs, considering the essence of vehicle multidirectional motion coupling, the multidirectional motion coupling control law is designed based on the multi degrees of freedom vehicle dynamic model. Based on the 8-DOF vehicle dynamic model, combined with sliding mode algorithm, the path tracking control law, vehicle speed tracking control law, yaw stability control law, and active suspension control law are designed to realize vehicle longitudinal force, wheel angle, additional yaw moment and additional roll moment control. In this paper, the method of controlling longitudinal force, wheel angle, yaw control torque, and roll control torque simultaneously based on the 8-DOF vehicle dynamics model is called the multidirectional motion coupling control method.
3. The effectiveness and superiority of the proposed extreme speed estimation method based on dynamic boundary and the multidirectional motion coupling control law design method based on 8-DOF vehicle dynamic model are verified by the simulation of double-line-shifting and serpentine driving under different speeds, different road adhesion coefficients, and different road curvatures.

The follow-up content of this paper is organized as follows: “Dynamic-boundary-based extreme speed estimation of DDAVS”, combined with the improved dynamic boundary, designs the extreme speed estimation algorithm. “Design of multidirectional motion coupling control law”, combined with 8-DOF DDAVs vehicle dynamics model design multidirectional motion coupling control law. In “Demonstrative example”, the Multidirectional Motion Coupling Control System (MMCCS) designed based on the proposed method is applied to an example DDAV model. Through simulation analysis, the effectiveness and superiority of the proposed methods under extreme conditions are verified. “Conclusion” summarizes and prospects the research content of this paper.

## Dynamic-boundary-based EXTREME SPEED estimation of DDAVS

The purpose of extreme speed estimation is to identify the extreme speed of stable driving of DDAVs according to driving conditions. Furthermore, combined with the speed control strategy, the vehicle speed is controlled below the extreme speed to avoid sideslip, tail flick, rollover, and longitudinal slip. The focus of extreme speed estimation is how to identify the steady state of DDAVs and find the speed when the tire force reaches saturation under extreme conditions. The dynamic boundary takes yaw rate and sideslip angle as the description objects, which is an effective tool for evaluating the stability of DDAVs^29^. The unstable boundary in the dynamic boundary describes the state of instability when the lateral force of DDAVs tire reaches saturation^29^. From the meaning of unstable boundary in dynamic boundary, it can be known that the vehicle speed is extreme speed when the vehicle dynamic response reaches the unstable boundary. Yaw rate, sideslip angle and roll angle are important parameters to describe the stable state of the vehicle during movement. To describe the driving stability of DDAVs more comprehensively, roll angle is introduced as the boundary to describe the vehicle roll stability based on the existing dynamic boundary^29^. For ease of description, the existing dynamic boundary is called the two-factor (2-FAC) dynamic boundary, and the improved dynamic boundary is called the three-factor (3-FAC) dynamic boundary. The extreme speed estimation algorithm is designed using the 3-FAC dynamic boundary.

### Dynamic boundary improvement

The derivation process of the 3-FAC dynamic boundary is similar to that of the 2-FAC dynamic boundary, and only the differences are described below. For more details, please refer to our previous research work^29^.

3-FAC dynamic boundary construction depends on the 3-DOF vehicle dynamics model^30^ including yaw rate, sideslip angle, and roll angle.

1 $${\mathcalligra{m}}\left({\dot{V}}_{\mathcal{Y}}+{V}_{\mathcal{X}}\gamma \right)+\left({\mathrm{am}}_{\mathrm{f}}-{\mathrm{bm}}_{\mathrm{r}}\right)\dot{\upgamma }+{\mathrm{m}}_{\mathrm{b}}{h}_{\mathrm{b}}\ddot{\phi }={F}_{\mathcal{Y}f}\mathrm{cos}\delta +{F}_{\mathcal{Y}r},$$

2 $$\left({\mathrm{am}}_{\mathrm{f}}-{\mathrm{bm}}_{\mathrm{r}}\right)\left({\dot{V}}_{\mathcal{Y}}+{V}_{\mathcal{X}}\gamma \right)+{I}_{z}\dot{\gamma }=a{F}_{\mathcal{Y}f}\mathrm{cos}\delta -b{F}_{\mathcal{Y}r},$$

3 $${{I}_{x}\ddot{\phi }+{\mathrm{m}}_{\mathrm{b}}{h}_{\mathrm{b}}\left({\dot{V}}_{\mathcal{Y}}+{V}_{\mathcal{X}}\gamma \right)+{I}_{xz}\dot{\gamma }=\Delta }_{\mathrm{df}}{F}_{\mathcal{Y}f}\mathrm{cos}\delta +{\Delta }_{\mathrm{dr}}{F}_{\mathcal{Y}r}-{D}_{\varnothing }\dot{\phi }-\left({C}_{\varnothing }-{m}_{b}g{h}_{b}\right)\phi ,$$

where $\mathcalligra{m}$ is the mass of the entire vehicle; ${V}_{\mathcal{X}}$ is the longitudinal vehicle speed; ${V}_{\mathcal{Y}}$ is the lateral vehicle speed; $\gamma$ is the yaw rate; $\delta$ is the average turning angle of the front wheels; a and b are the distances from the front and rear axles to the center of mass, respectively; $V$ is the vehicle speed; ${\mathrm{m}}_{\mathrm{f}}$ and ${\mathrm{m}}_{\mathrm{r}}$ are the front suspension unsprung mass and rear suspension unsprung mass; ${I}_{z}$ is the vehicle yaw motion moment of inertia; ${I}_{x}$ is the moment of inertia of body roll motion; ${I}_{xz}$ is the inertial product of roll and yaw motions; $\phi$ is roll angle; ${D}_{\varnothing }$ is the roll damping coefficient; ${C}_{\varnothing }$ is the roll stiffness; ${\mathrm{m}}_{\mathrm{b}}$ is the vehicle sprung mass; ${h}_{\mathrm{b}}$ is the distance between the mass center of spring load and the roll axis; $g$ is the acceleration of gravity; ${\Delta }_{\mathrm{df}}$ is the front tire lateral offset caused by the unit roll angle; ${\Delta }_{\mathrm{dr}}$ is the lateral offset of rear tire caused by unit roll angle; ${F}_{\mathcal{Y}f}$ and ${F}_{\mathcal{Y}r}$ are the lateral forces on the front wheel and rear wheel.

To improve the real-time performance of DDAVs motion control algorithm, the vehicle dynamics model is further simplified as follows: assuming that the front wheel angle is small, then $\mathrm{cos}\delta \approx 1$^29^; assuming that the body roll axis is parallel to the ground, then ${\Delta }_{\mathrm{df}}=0$, ${\Delta }_{\mathrm{dr}}=0$^30^. The simplified 3-DOF vehicle model is as follows:

4 $$\mathcalligra{m}\left({\dot{V}}_{\mathcal{Y}}+{V}_{\mathcal{X}}\gamma \right)+\left({\mathrm{am}}_{\mathrm{f}}-{\mathrm{bm}}_{\mathrm{r}}\right)\dot{\upgamma }+{\mathrm{m}}_{\mathrm{b}}{h}_{\mathrm{b}}\ddot{\phi }={F}_{\mathcal{Y}f}+{F}_{\mathcal{Y}r},$$

5 $$\left({\mathrm{am}}_{\mathrm{f}}-{\mathrm{bm}}_{\mathrm{r}}\right)\left({\dot{V}}_{\mathcal{Y}}+{V}_{\mathcal{X}}\gamma \right)+{I}_{z}\dot{\gamma }=a{F}_{\mathcal{Y}f}-b{F}_{\mathcal{Y}r},$$

6 $${I}_{x}\ddot{\phi }+{\mathrm{m}}_{\mathrm{b}}{h}_{\mathrm{b}}\left({\dot{V}}_{\mathcal{Y}}+{V}_{\mathcal{X}}\gamma \right)+{I}_{xz}\dot{\gamma }=-{D}_{\varnothing }\dot{\phi }-\left({C}_{\varnothing }-{m}_{b}g{h}_{b}\right)\phi .$$

The ${F}_{\mathcal{Y}f}$ and ${F}_{\mathcal{Y}r}$ in Eqs. (4)–(6) are provided by the simplified Dugoff tire model^29^.

The dynamic boundary consists of stable boundary and unstable boundary. The stability boundary is composed of the state evaluation parameters when the vehicle response reaches steady state, which describes the vehicle in a stable and controllable state. When the vehicle dynamic response reaches steady state, $\gamma$ and $\phi$ are constant, so $\dot{\gamma }=0$; $\dot{\phi }=\ddot{\phi }=0$; ${\dot{V}}_{\mathcal{Y}}=0$. Combined Eqs. (4)–(6), the expressions of yaw rate, sideslip angle, and roll angle are as follows:

7 $${\gamma }_{s\mu }=\frac{{V}_{\mathcal{X}}}{L\left(1+{K}_{\mu }{V}_{\mathcal{X}}^{2}\right)} \delta ,$$

8 $${\beta }_{s\mu }=\frac{b-\frac{{V}_{\mathcal{X}}^{2}\left(am+{\mathrm{bm}}_{\mathrm{r}}-{\mathrm{am}}_{\mathrm{f}}\right)}{{C}_{\mathcal{Y}f}f\left({\upsigma }_{r}\right)L}}{L\left(1+{K}_{\mu }{V}_{\mathcal{X}}^{2}\right)} \delta ,$$

9 $${\phi }_{s\mu }=\frac{{V}_{\mathcal{X}}^{2}{m}_{b}{h}_{b}}{L\left(1+{K}_{\mu }{V}_{\mathcal{X}}^{2}\right)\left({m}_{b}g{h}_{b}-{C}_{\varnothing }\right)} \delta ,$$

where ${K}_{\mu }=\frac{1}{{L}^{2}}\left[m\left(\frac{a}{{C}_{\mathcal{Y}r}f\left({\upsigma }_{r}\right)}-\frac{b}{{C}_{\mathcal{Y}f}f\left({\upsigma }_{f}\right)}\right)+\left({\mathrm{bm}}_{\mathrm{r}}-{\mathrm{am}}_{\mathrm{f}}\right)\left(\frac{1}{{C}_{\mathcal{Y}r}f\left({\upsigma }_{r}\right)}+\frac{1}{{C}_{\mathcal{Y}f}f\left({\upsigma }_{f}\right)}\right)\right]$; $L$ is the axis distance; ${C}_{\mathcal{Y}f}$ is the front tire corner stiffness; ${C}_{\mathcal{Y}r}$ is the rear tire corner stiffness; $f\left({\upsigma }_{i}\right)$ is the parameter in the simplified Dugoff tire model^29^, which includes the road adhesion coefficient $\mu$. ${K}_{\mu }$ is the stability factor, which is an important parameter to characterize the steady-state response of the vehicle. Compared with the traditional stability factor^31^, the stability factor considers the influence of vehicle sprung mass and road adhesion characteristics. ${\gamma }_{s\mu }$, ${\beta }_{s\mu }$ and ${\phi }_{s\mu }$ jointly constitute the stable boundary of the 3-FAC dynamic boundary proposed in this paper.

The unstable boundary in the 3-FAC dynamic boundary is composed of the state evaluation parameters of side slip, tail flick, and rollover, which describes the state that the vehicle may be out of control. The adhesion force provided by tires is limited during driving. When the tire force required for stable motion exceeds the maximum adhesion force generated by tires, the vehicle will slide on the ground plane, resulting in dangerous phenomena such as longitudinal slip, side slip, and tail flick. Therefore, it is an effective method to avoid vehicle instability to clarify the maximum friction force generated by the tire and control the tire force required to maintain the stable movement of the vehicle.

The maximum adhesion force generated by tires during vehicle driving satisfies the following relationship:

10 $${F}_{t}\le \mu mg.$$

The longitudinal and lateral forces on the ground of the vehicle satisfy the following relations:

11 $${{F}_{t}}^{2}={{F}_{x}}^{2}+{{F}_{y}}^{2}.$$

So:

12 $${F}_{y}=m{a}_{\mathcal{Y}}=\sqrt{{{F}_{t}}^{2}-{{F}_{x}}^{2}}.$$

According to the vehicle driving equations^31^:

13 $${F}_{x}=m{a}_{x}+mg\left({i}_{0}+f\right)+\frac{{C}_{D}A{V}_{x}}{21.15},$$

where ${a}_{x}$ is the longitudinal acceleration, ${i}_{0}$ is the longitudinal slope, $f$ is the rolling resistance coefficient, ${C}_{D}$ is the air resistance coefficient, $A$ is the windward area.

Combining Eqs. (10)–(13), the following relationship can be obtained:

14 $$\left|{a}_{\mathcal{Y}}\right|\le \sqrt{{\left(\mu g\right)}^{2}-{\left({a}_{x}+g\left(i+f\right)+\frac{{C}_{D}A{V}_{x}}{21.15m}\right)}^{2}}.$$

The lateral acceleration during vehicle driving satisfies the following relationship:

15 $${a}_{\mathcal{Y}}={V}_{\mathcal{X}}\gamma +{\dot{V}}_{\mathcal{Y}}.$$

According to the definition of sideslip angle $\beta$, ${V}_{\mathcal{Y}}={V}_{\mathcal{X}}\mathrm{tan}\beta$. Generally, the value of $\left|\beta \right|$ is small, then $\mathrm{tan}\beta \approx \beta$^32^. So, Eq. (15) can be rewritten as:

16 $${a}_{\mathcal{Y}}={V}_{\mathcal{X}}\gamma +{\dot{V}}_{\mathcal{X}}\beta +{V}_{\mathcal{X}}\dot{\beta }.$$

In general, the latter two values of Eq. (16) are very small relative to the first item, so Eq. (16) can be rewritten as^32^:

17 $${a}_{\mathcal{Y}}={V}_{\mathcal{X}}\gamma $$

Therefore, combined with Eqs. (14) and (17), the upper limit of $\gamma$ is:

18 $$\left|{\gamma }_{max}\right|=\frac{\sqrt{{\left(\mu g\right)}^{2}-{\left({a}_{x}+g\left(i+f\right)+\frac{{C}_{D}A{V}_{x}}{21.15m}\right)}^{2}}}{\left|{V}_{\mathcal{X}}\right|}.$$

By analyzing Eqs. (7) and (8), it can be seen that ${\gamma }_{s\mu }$ and ${\beta }_{s\mu }$ have the following relationship: ${\beta }_{s\mu }={\gamma }_{s\mu }\left(\frac{b}{{V}_{\mathcal{X}}}-\frac{{V}_{\mathcal{X}}\left(am+{\mathrm{bm}}_{\mathrm{r}}-{\mathrm{am}}_{\mathrm{f}}\right)}{{C}_{\mathcal{Y}f}f\left({\upsigma }_{r}\right)L}\right)$, so the upper limit of $\beta$ is:

19 $${\beta }_{max}=\frac{\sqrt{{\left(\mu g\right)}^{2}-{\left({a}_{x}+g\left(i+f\right)+\frac{{C}_{D}A{V}_{x}}{21.15m}\right)}^{2}}}{{V}_{\mathcal{X}}}\left(\frac{b}{{V}_{\mathcal{X}}}-\frac{{V}_{\mathcal{X}}\left(am+{\mathrm{bm}}_{\mathrm{r}}-{\mathrm{am}}_{\mathrm{f}}\right)}{{C}_{\mathcal{Y}f}f\left({\upsigma }_{r}\right)L}\right).$$

The roll instability during vehicle driving is mainly manifested as rollover or side slip, where the force equilibrium equation of rollover critical state is as follows:

20 $${m}_{b}{h}_{c}\left|{a}_{y}\right|+\left({m}_{f}+{m}_{r}\right){h}_{d}\left|{a}_{y}\right|={m}_{b}g\left(\frac{{d}_{f}+{d}_{r}}{4}-\mathrm{\varnothing }{h}_{b}\right)+mg{h}_{c}\alpha +\frac{{m}_{f}+{m}_{r}}{2}g\frac{{d}_{f}+{d}_{r}}{2},$$

where ${h}_{c}$ is the high centroid of the vehicle, ${h}_{d}$ is the high centroid of the unsprung mass, $\alpha$ is the transverse slope of the road, ${d}_{f}$ is the front wheel distance; ${d}_{r}$ is the rear wheel distance.

From Eq. (20), the maximum roll angle under the rollover critical state is:

21 $$\left|{\mathrm{\varnothing }}_{max1}\right|=\frac{mg\left(\frac{{d}_{f}+{d}_{r}}{4}+{h}_{c}\alpha \right)-\left|{a}_{y}\right|\left[{h}_{d}\left({m}_{f}+{m}_{r}\right)+{{h}_{c}m}_{b}\right]}{{m}_{b}g{h}_{a}}.$$

The critical state force equilibrium equation of side slip is as follows:

22 $${m}_{b}{h}_{c}\left|{a}_{ymax}\right|+\left({m}_{f}+{m}_{r}\right){h}_{d}\left|{a}_{ymax}\right|={m}_{b}g\left(\frac{{d}_{f}+{d}_{r}}{4}-\mathrm{\varnothing }{h}_{a}\right)+mg{h}_{c}\alpha -\left({F}_{zfx}+{F}_{zrx}\right)\frac{{d}_{f}+{d}_{r}}{2},$$

where ${F}_{zfx}$ and ${F}_{zrx}$ are the vertical forces of the front tire and the rear tire. When ${a}_{ymax}$ points to the right side of the vehicle, ${F}_{zfx}={F}_{zfl}$, ${F}_{zrx}={F}_{zrl}$. When ${a}_{ymax}$ points to the left side of the vehicle, ${F}_{zfx}={F}_{zfr}$, ${F}_{zrx}={F}_{zrr}$.

The maximum roll angle of the vehicle under the critical condition of side slip obtained by Eq. (22) is:

23 $$\left|{\mathrm{\varnothing }}_{max2}\right|=\frac{{m}_{b}g\frac{{d}_{f}+{d}_{r}}{4}+mg{h}_{c}\alpha -\left({F}_{zfx}+{F}_{zrx}\right)\frac{{d}_{f}+{d}_{r}}{2}}{{m}_{b}g{h}_{a}}-\frac{\left|{a}_{\mathcal{Y}max}\right|\left[{h}_{d}\left({m}_{f}+{m}_{r}\right)+{{h}_{c}m}_{b}\right]}{{m}_{b}g{h}_{a}}.$$

Combining Eqs. (21) and (23), the maximum allowable roll angle during driving is:

24 $${\mathrm{\varnothing }}_{max}=\mathrm{min}\left(\left|{\mathrm{\varnothing }}_{max1}\right| , \left|{\mathrm{\varnothing }}_{max2}\right|\right).$$

The ${\gamma }_{max}$, ${\beta }_{max}$ and ${\mathrm{\varnothing }}_{max}$ obtained from the above analysis can comprehensively reflect the vehicle stability state under tail flick, side slip, and rollover conditions, namely, the unstable boundary of the 3-FAC dynamic boundary.

### Design of extreme speed estimation algorithm

The focus of extreme speed estimation is how to find the speed when the tire force is about to reach saturation under extreme conditions. From the meaning of unstable boundary, it can be seen that when the vehicle dynamic response reaches the unstable boundary, the speed is extreme speed. At the same time, the function of MMCCS is to improve the driving stability of DDAVs, so that the vehicle's motion response is far away from the unstable boundary, or can still run stably when approaching the unstable boundary. Therefore, the vehicle speed when the DDAVs motion response is close to the unstable boundary and can run stably is the safe extreme speed to be found. At this time, the stable boundary and the unstable boundary are equal. Therefore, the extreme speed solving equations are constructed from the expression of the 3-FAC dynamic boundary as follows:

25 $$\left\{\begin{array}{c}f\left({V}_{x\_max\gamma }\right)={\gamma }_{max}-{\gamma }_{s\mu }=0\\ f\left({V}_{x\_max\beta }\right)={\beta }_{max}-{\beta }_{s\mu }=0\\ f\left({V}_{x\_max\phi }\right)={\phi }_{max}-{\phi }_{s\mu }=0\\ f\left({V}_{x\_max}\right)=min\left({V}_{x\_max\gamma } ,{V}_{x\_max\beta } ,{V}_{x\_max\phi }\right),\end{array}\right.$$

where ${V}_{x\_max\gamma }$ is the extreme speed estimated according to the yaw rate; ${V}_{x\_max\beta }$ is the extreme speed estimated according to sideslip angle; ${V}_{x\_max\phi }$ is the extreme speed estimated according to the roll angle; ${V}_{x\_max}$ is extreme speed.

Equations (25) contain high-order polynomials, which cannot directly obtain effective analytical solutions. Therefore, the problem of solving the equations is transformed into an optimization problem, and the objective function for solving extreme speed is constructed by combining Eq. (25) as follows:

26 $$\left\{\begin{array}{c}f\left({V}_{x\_max\gamma }\right)=\mathrm{min}\left(\left|{\gamma }_{max}-{\gamma }_{s\mu }\right|\right)\\ f\left({V}_{x\_max\beta }\right)=\mathrm{min}\left(\left|{\beta }_{max}-{\beta }_{s\mu }\right|\right)\\ f\left({V}_{x\_max\phi }\right)=\mathrm{min}\left(\left|{\phi }_{max}-{\phi }_{s\mu }\right|\right)\\ f\left({V}_{x\_max}\right)=min\left({V}_{x\_max\gamma } ,{V}_{x\_max\beta } ,{V}_{x\_max\phi }\right).\end{array}\right.$$

**Figure 1:**
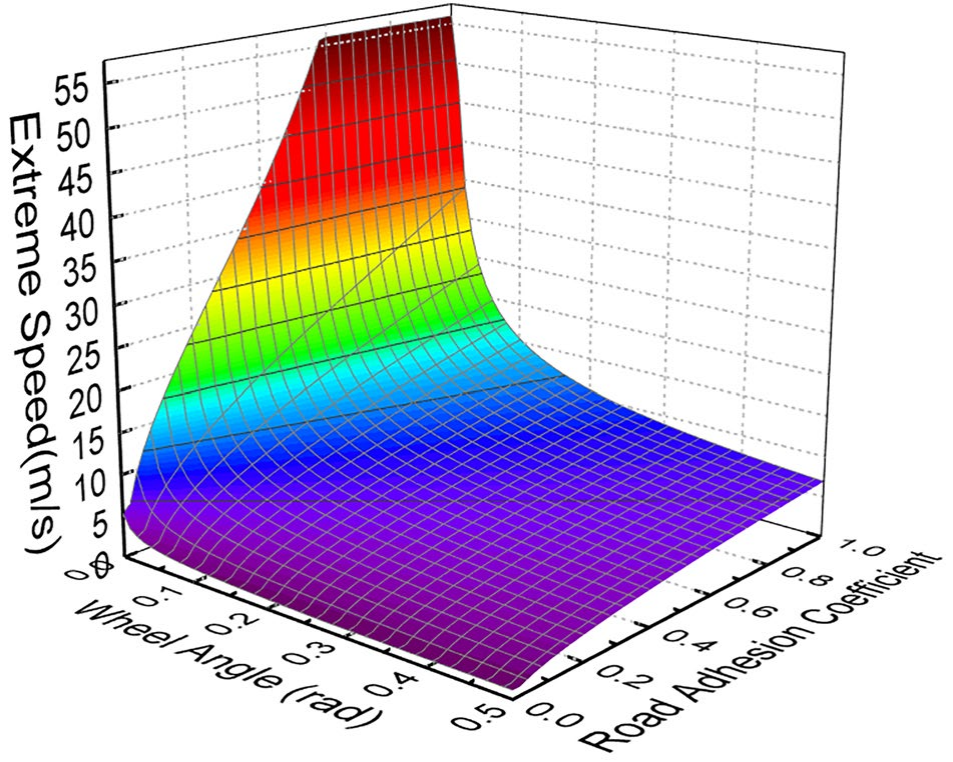
The extreme speed at different road adhesion coefficients and wheel angles.

Assume that the road is level and the longitudinal acceleration is zero when the extreme speed is reached. Taking Eq. (26) as the objective function, combined with the physical constraints of each parameter, the extreme speed under each working condition can be obtained by calling the optimization solution function in the Matlab optimization toolbox. Figure 1 shows the extreme speed at different road adhesion coefficients and different wheel angles.

Although the extreme speed of DDAVs at different road adhesion coefficients and different wheel angles has been obtained through the above research, the extreme speed tracking control cannot be realized only by the extreme speed shown in Fig. 1. Because, when the vehicle is running under extreme conditions, such as double-line-shifting condition and continuous turning condition, the wheel angle is continuously changing, and extreme speed also follows the change. However, in order to drive safely and reduce the discomfort of passengers, it is generally hoped that the vehicle can slow down to the safe speed range before entering the extreme condition, and the speed can remain stable in the extreme condition. Therefore, based on the above research, combined with the high-precision map of DDAVs, the extreme speed preview method is proposed. Firstly, the road curvature is obtained according to the high-precision map, and the road curvature radius ${R}_{r}$ is calculated on this basis. According to the kinematic relationship during driving, the average rotation angle of the front wheel is as follows^31^:

27 $$\delta =\mathrm{tan}\frac{L}{{R}_{r}}.$$

**Figure 2:**
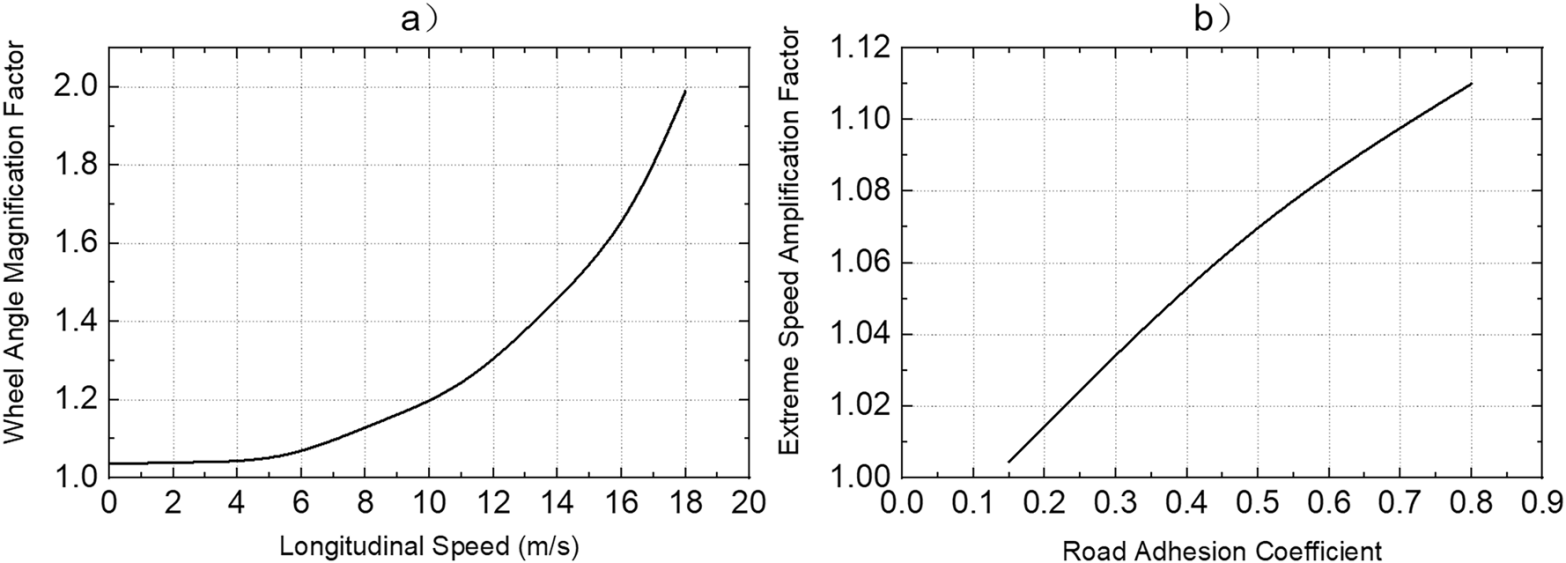
(**a**) Relationship between average wheel angle magnification factor ${K}_{\delta }$ and longitudinal speed; (**b**) Relationship between extreme speed magnification factor ${K}_{v}$ and road adhesion coefficient.

The relationship between ${R}_{r}$ and $\delta$ described by Eq. (27) is only applicable when the vehicle is in the range of kinematic response. The dynamic characteristics of a vehicle must be taken into account when driving under extreme conditions, so the Eq. (27) is rewritten as:

28 $$\delta ={K}_{\delta }\mathrm{tan}\frac{L}{{R}_{r}},$$

where ${K}_{\delta }$ is the average angular magnification factor of the front wheels. According to the daily driving experience, ${K}_{\delta }$ is closely related to vehicle speed. This paper also focuses on the relationship between ${K}_{\delta }$ and road adhesion coefficient. Through the simulation analysis under different speeds, different road adhesion coefficients and different road curvatures, the relationship between ${K}_{\delta }$ and speed is obtained as shown in Fig. 2a. The effect of road adhesion coefficient on ${K}_{\delta }$ is small when the dynamic response of DDAVs does not exceed the unstable boundary. This is because when the vehicle dynamic response does not exceed the unstable boundary, the adhesion of the ground to the wheels does not reach the limit and is not limited by road adhesion. Because the safety extreme speed ${V}_{x\_maxs}$ studied in this paper is the response of the vehicle in an unstable boundary, the influence of road adhesion coefficient is ignored, and the final value of ${K}_{\delta }$ is shown in Fig. 2a. To calculate the safety extreme speed, the optimization objective function is designed as follows:

29 $${V}_{x\_maxs}=\mathrm{min}\left(\left|{V}_{x\_\mathit{max} n}-{V}_{x\_\mathit{max} n-1}\right|\right),$$

where ${V}_{x\_\mathrm{max} n-1}$ is obtained from the data mapping table of ${V}_{x\_max}$ shown in Fig. 1; ${V}_{x\_\mathit{max} n}$ is calculated as follows: V ${V}_{x\_\mathit{max} n-1}$ according to the data mapping table of ${K}_{\delta }$ shown in Fig. 2a, the average angle magnification factor of the front wheels is obtained, and the corresponding $\delta$ is calculated. Then ${V}_{x\_\mathit{max} n}$ is obtained through the data mapping table of ${V}_{x\_max}$ with the input of the $\delta$ and the current road adhesion coefficient. When $n=1$, the $\delta$ calculated by Eq. (27) and the current road adhesion coefficient are used as inputs, and the ${V}_{x\_\mathit{max} 1}$ is obtained by checking the mapping table. This constitutes the iterative solution process of ${V}_{x\_maxs}$ through the mapping table of data in Figs. 1 and 2a. The safety extreme speed under various road curvatures and road adhesion coefficients can be obtained by calling the optimization solution function in the Matlab optimization toolbox.

The safety extreme speed obtained by the Eq. (29) is to ensure that the dynamic response of DDAVs is completely in an unstable boundary. However, the unstable boundary is the mathematical description for identifying vehicle stability proposed in this paper, and there are still many factors that are not considered. It is found in the simulation that even if the dynamic response slightly exceeds the unstable boundary, the vehicle is still in a controllable range in the driving process of DDAVs. To give full play to the control potential of MMCCS and improve the efficiency of automobile transportation, ${V}_{x\_maxs}$ is amplified. The final extreme speed after amplification is as follows:

30 $${V}_{x\_maxf}={K}_{v}{V}_{x\_maxs},$$

where ${K}_{v}$ is the extreme speed amplification factor. Through simulation analysis under various working conditions, it is concluded that when DDAVs reach the ultimate extreme speed, the relationship between ${K}_{v}$ and road adhesion coefficient is shown in Fig. 2b, and the change of front wheel average angle has little effect on ${K}_{v}$.

According to the road adhesion coefficient and road curvature, the final extreme speed of each point under the path can be obtained. Then, according to the curvature and curvature change rate of the path, the road bending can be judged to determine the bending and out-bending locations of DDAVs. The vehicle speed is controlled below the ultimate extreme speed before entering the bend or before entering the low adhesion road to ensure the safe passage of the vehicle. The final expected speed of DDAVs is as follows:

31 $${V}_{x\_f}=\mathrm{min}\left(\left|{V}_{x\_maxf}\right|,\left|{V}_{x\_p}\right|\right),$$

where ${V}_{x\_p}$ is the expected speed given by the vehicle motion control decision layer.

## Design of multidirectional motion coupling control law

The focus of control layer design is to design multidirectional motion coupling control law based on multi degrees of freedom vehicle dynamics model and sliding mode algorithm. The 8-DOF vehicle dynamics is built to control the vehicle longitudinal force, wheel angle, additional yaw moment, and additional roll moment. The 8-DOF vehicle dynamic model including DDAVs longitudinal motion, lateral motion, yaw motion, roll motion, and four independent wheels rotation is as follows:

32 $$\mathcalligra{m}\left({\dot{V}}_{\mathcal{X}}+{V}_{\mathcal{Y}}\gamma \right)+{\mathrm{m}}_{\mathrm{b}}{h}_{\mathrm{b}}\gamma \dot{\phi }=\left({F}_{\mathcal{X}fr}+{F}_{\mathcal{X}fl}\right)\mathrm{cos}\delta -\left({F}_{\mathcal{Y}fr}+{ F}_{\mathcal{Y}fl}\right)\mathrm{sin}\delta +{F}_{\mathcal{X}rl}+{F}_{\mathcal{X}rr}-mg\left(i+f\right)-\frac{{C}_{D}A{V}_{x}}{21.15}+\Delta {F}_{\mathcal{X}},$$

33 $$\mathcalligra{m}\left({\dot{V}}_{\mathcal{Y}}+{V}_{\mathcal{X}}\gamma \right)+\left({\mathrm{am}}_{\mathrm{f}}-{\mathrm{bm}}_{\mathrm{r}}\right)\dot{\upgamma }+{\mathrm{m}}_{\mathrm{b}}{h}_{\mathrm{b}}\ddot{\phi }=\left({F}_{\mathcal{Y}fr}+{F}_{\mathcal{Y}fl}\right)\mathrm{cos}\delta +\left({F}_{\mathcal{X}fr}+{ F}_{\mathcal{X}fl}\right)\mathrm{sin}\delta +{F}_{\mathcal{Y}rl}+{F}_{\mathcal{Y}rr},$$

34 $$\left({\mathrm{am}}_{\mathrm{f}}-{\mathrm{bm}}_{\mathrm{r}}\right)\left({\dot{V}}_{\mathcal{Y}}+{V}_{\mathcal{X}}\gamma \right)+{I}_{z}\dot{\gamma }=a\left({F}_{\mathcal{Y}fr}+{F}_{\mathcal{Y}fl}\right)\mathrm{cos}\delta +\frac{{d}_{f}}{2}\left({F}_{\mathcal{Y}fl}-{F}_{\mathcal{Y}fr}\right)\mathrm{sin}\delta - b\left({F}_{\mathcal{Y}rl}+{F}_{\mathcal{Y}rr} \right)+ \frac{{d}_{f}}{2}\left({F}_{\mathcal{X}fr}-{F}_{\mathcal{X}fl}\right)\mathrm{cos}\delta +a\left({F}_{\mathcal{X}fr}+{F}_{\mathcal{X}fl}\right)\mathrm{sin}\delta +{\frac{{d}_{r}}{2}\left({F}_{\mathcal{X}rr}-{F}_{\mathcal{X}rl}\right)+\Delta M}_{z},$$

35 $${I}_{x}\ddot{\phi }+{\mathrm{m}}_{\mathrm{b}}{h}_{\mathrm{b}}\left({\dot{V}}_{\mathcal{Y}}+{V}_{\mathcal{X}}\gamma \right)+{I}_{xz}\dot{\gamma }={\Delta }_{\mathrm{df}}\left({F}_{\mathcal{Y}fr}+{F}_{\mathcal{Y}fl}\right)\mathrm{cos}\delta +{\Delta }_{\mathrm{dr}}\left({F}_{\mathcal{Y}rr}+{F}_{\mathcal{Y}rl}\right)-{D}_{\varnothing }\dot{\phi }-\left({C}_{\varnothing }-{m}_{b}g{h}_{b}\right)\phi +\Delta {M}_{\mathcal{X}},$$

36 $${J}_{w}{\dot{\omega }}_{ij}={T}_{dij}-{T}_{bij}-{F}_{\mathcal{X}ij}R,$$

where ${F}_{\mathcal{X}ij}$ is the tire longitudinal force; ${F}_{\mathcal{Y}ij}$ is the tire lateral force; ${\Delta M}_{z}$ is the yaw control torque; $\Delta {F}_{\mathcal{X}}$ is the longitudinal control force; $\Delta {M}_{\mathcal{X}}$ is the roll control torque; ${J}_{w}$ is the wheel inertia; ${\omega }_{ij}$ is the wheel speed; ${T}_{dij}$ is the wheel drive torque; ${T}_{bij}$ is the wheel braking torque; $R$ is the tire rolling radius. (in the expression form ${x}_{ij}$, footmark $i$ denotes the front wheel or rear wheel, $j$ denotes the left wheel or right wheel). The tire force in the model is calculated by the Dugoff nonlinear tire model^33^. The tire model is an analytical model derived from the force balance relationship. This model has fewer self-defined parameters and can better express the nonlinear characteristics of tires, which is widely used in vehicle dynamic motion control^29^.

### PFC control law design

Design sliding mode:

37 $${s}_{P}=\dot{{e}_{P}}+{c}_{P}{e}_{P},$$

where ${c}_{P}$ is an adjustable parameter and ${c}_{P}>$ 0; ${e}_{P}$ is the path following error.

Exponential approach law is adopted:

38 $$\dot{{s}_{P}}=-{\varepsilon }_{p}\cdot sgn\left({s}_{P} \right)-{k}_{p}{s}_{P},$$

where ${\varepsilon }_{p}$ and ${k}_{p}$ are adjustable parameters.

When the modeling uncertainty and interference are large, the switching term gain ${\varepsilon }_{p}$ is required to be large, which will cause great chattering. To improve chattering, the saturation function $sat\left({s}_{P}\right)$ is usually used to replace the sign function $sgn\left({s}_{P}\right)$^28^. The saturation function expression is as follows:

39 $$sat\left({s}_{P} \right)=\left\{\begin{array}{c}1 {s}_{P}>\Delta \\ {k}_{ps}{s}_{P} \left|{s}_{P}\right|\le \Delta \\ -1 {s}_{P}<-\Delta ,\end{array}\right.$$

where ${k}_{ps}$ is an adjustable parameter, and ${k}_{ps}=\frac{1}{\Delta }$.

Therefore, Eq. (38) is rewritten as:

40 $$\dot{{s}_{P}}=-{\varepsilon }_{p}\cdot sat\left({s}_{P} \right)-{k}_{p}{s}_{P}.$$

The change rate of DDAVs path tracking error is:

41 $$\dot{{e}_{P}}={V}_{\mathcal{X}}\mathrm{sin}{\psi }_{R}+{V}_{\mathcal{Y}}\mathrm{cos}{\psi }_{R},$$

where ${\psi }_{R}$ is the yaw angle of the vehicle longitudinally relative to the path. Since ${\psi }_{R}$ is generally small, $\mathrm{sin}{\psi }_{R}\approx {\psi }_{R}$, $\mathrm{cos}{\psi }_{R}\approx 1$, so (41) is rewritten as:

42 $$\dot{{e}_{P}}={V}_{\mathcal{X}}{\psi }_{R}+{V}_{\mathcal{Y}}.$$

According to Eq. (33), the change rate of lateral speed is as follows:

43 $${\dot{V}}_{\mathcal{Y}}=\frac{\left({F}_{\mathcal{Y}fr}+{F}_{\mathcal{Y}fl}\right)\mathrm{cos}\delta +\left({F}_{\mathcal{X}fr}+{ F}_{\mathcal{X}fl}\right)\mathrm{sin}\delta }{m}+\frac{{F}_{\mathcal{Y}rl}+{F}_{\mathcal{Y}rr}-\left({\mathrm{am}}_{\mathrm{f}}-{\mathrm{bm}}_{\mathrm{r}}\right)\dot{\upgamma }-{\mathrm{m}}_{\mathrm{b}}{h}_{\mathrm{b}}\ddot{\phi }}{m}-{V}_{\mathcal{X}}\gamma .$$

In the driving process, $\delta$ is generally small, so $\mathrm{sin}\delta \approx \delta$ and $\mathrm{cos}\delta \approx 1$. Similarly, $\mathrm{tan}\delta \approx \delta$, the tire lateral force in Dugoff tire model is simplified as follows:

44 $${F}_{\mathcal{Y}ij}={C}_{\mathcal{Y}ij}\frac{{\alpha }_{ij}}{1+{\uplambda }_{ij}}f\left({\upsigma }_{ij}\right),$$

where ${C}_{\mathcal{Y}ij}$ is the tire cornering stiffness; ${\uplambda }_{ij}$ is the wheel slip ratio; ${\alpha }_{ij}$ is the tire cornering angle; the expression of $f\left({\upsigma }_{ij}\right)$ can be found in Dugoff tire model^33^.

Combined with Eqs. (37), (40), (42)–(44), and tire cornering calculation equations^34^, the average front wheel cornering angle δ sliding mode control law is calculated as follows:

45 $$\delta =\frac{-m\left\{{V}_{x}\dot{{\psi }_{R}}+{S}_{pr}{k}_{p}+{c}_{p}{\dot{e}}_{p}+{\varepsilon }_{p}sat+{\dot{V}}_{x}{\psi }_{R}-A\right\}}{{C}_{yflp}+{C}_{yfrp}+{ F}_{xfl}+{ F}_{xfr}},$$

$$A=\frac{\dot{\gamma }\left({L}_{f}{m}_{f}-{L}_{r}{m}_{r}\right)+{C}_{yflp}actan\left(\frac{{V}_{y}+{L}_{f}\gamma }{{V}_{x}-\frac{{d}_{f}\gamma }{2}}\right)+{C}_{yfrp}actan\left(\frac{{V}_{y}+{L}_{f}\gamma }{{V}_{x}+\frac{{d}_{f}\gamma }{2}}\right)}{m}+\frac{{C}_{yrlp}actan\left(\frac{{V}_{y}-{L}_{r}\gamma }{{V}_{x}-\frac{{d}_{f}\gamma }{2}}\right)+{C}_{yrrp}actan\left(\frac{{V}_{y}-{L}_{r}\gamma }{{V}_{x}+\frac{{d}_{f}\gamma }{2}}\right)+{V}_{x}\gamma m-\ddot{\phi }{h}_{b}{m}_{b}}{m},$$

where $${C}_{yijp}=\frac{{C}_{\mathcal{Y}ij}}{1+{\uplambda }_{ij}}f\left({\upsigma }_{ij}\right)$$.

### STC control law design

Design sliding mode:

46 $${s}_{s}=\dot{{e}_{s}}+{c}_{s}{e}_{s},$$

where ${c}_{s}$ is an adjustable parameter and ${c}_{s}>$ 0; ${e}_{s}$ is the speed tracking error.

Similarly, the saturation function $sat\left({s}_{s}\right)$ is used to replace the symbol function $sgn\left({s}_{s}\right)$^28^, and the exponential reaching rate is as follows:

47 $$\dot{{s}_{s}}=-{\varepsilon }_{s}\cdot sat\left({s}_{s} \right)-{k}_{s}{s}_{s},$$

where ${\varepsilon }_{s}$ and ${k}_{s}$ are adjustable parameters.

Combined with Eqs. (32), (46), and (47), the expected longitudinal force $\Delta {F}_{\mathcal{X}}$ sliding mode control law is calculated as follows:

48 $${F}_{xc}=\frac{{\ddot{V}}_{xd}-{V}_{x}-{\varepsilon }_{L}sat+{C}_{L}\left[{\dot{V}}_{xd}+B\right]}{\left(\frac{{C}_{L}}{m}+\frac{{K}_{1L}}{m}\right)}+\frac{{K}_{1L}\left[{\dot{V}}_{xd}-{C}_{L}\left({V}_{x}-{V}_{xd}\right)+B\right]}{\left(\frac{{C}_{L}}{m}+\frac{{K}_{1L}}{m}\right)},$$

$$B=\frac{\mathit{sin}\delta \left({F}_{yfl}+{F}_{yfr}\right)-{F}_{xrr}-cos\delta \left({F}_{xfl}+{F}_{xfr}\right)-{F}_{xrl}}{m}+\frac{\frac{\left(20{A}_{W}{C}_{D}{V}_{x}\right)}{423}+gm\left(ff+ii\right)+{V}_{y}\gamma m+\dot{\varphi }\gamma {h}_{a}{m}_{u}}{m}.$$

### YSC control law design

Design sliding mode:

49 $${s}_{Y}=\dot{{e}_{Y}}+{c}_{Y}{e}_{Y},$$

where ${c}_{Y}$ is an adjustable parameter and ${c}_{Y}>$ 0; ${e}_{Y}$ is the target tracking error. To enable YSC to control both yaw rate and Sideslip Angle within a safe range, ${e}_{Y}$ expression is designed as follows:

50 $${e}_{Y}=\left(\gamma -{\gamma }_{d\mu }\right)+\left(\beta -{\beta }_{d\mu }\right),$$

where ${\gamma }_{d\mu }$ is the target yaw rate; ${\beta }_{d\mu }$ is the target sideslip Angle. The function of YSC is to make the vehicle run in the stable region as far as possible. The control rules of YSC are as follows: when the vehicle runs in the quasi-stable region, the vehicle is controlled to enter the stable region; when the vehicle enters the unstable region, the vehicle is controlled to enter the quasi-stable region first. According to the above rules, the control objective of YSC based on the dynamic boundary is designed as follows:

51 $${\gamma }_{d\mu }=\left\{\begin{array}{c}{\gamma }_{s\mu }, \left|{\gamma }_{s\mu }\right|<\left|\gamma \right|<{\gamma }_{max}\\ {\gamma }_{max}\mathrm{sign}\left(\gamma \right), \left|\gamma \right|\ge {\gamma }_{max,}\end{array}\right.$$

52 $${\beta }_{d\mu }=\left\{\begin{array}{c}{\beta }_{s\mu }, \left|{\beta }_{s\mu }\right|<\left|\beta \right|<{\beta }_{max}\\ {\beta }_{max}\mathrm{sign}\left(\beta \right), \left|\beta \right|\ge {\beta }_{max.}\end{array}\right.$$

Similarly, the saturation function $sat\left({s}_{s}\right)$ is used to replace the symbol function $sgn\left({s}_{s}\right)$^28^, and the exponential reaching rate is as follows:

53 $$\dot{{s}_{Y}}=-{\varepsilon }_{Y}\cdot sat\left({s}_{Y} \right)-{k}_{Y}{s}_{Y},$$

where ${\varepsilon }_{Y}$ and ${k}_{Y}$ are adjustable parameters.

Combining Eqs. (34), (49), and (53), the expected additional yaw moment ${\Delta M}_{z}$ sliding mode control law is calculated as follows:

54 $${\Delta M}_{z}=\frac{\left({\ddot{\beta }}_{d}-\ddot{\beta }-\ddot{\gamma }+{\ddot{\gamma }}_{d}-{\varepsilon }_{Y}sat\right){I}_{z}+{C}_{Y}\left[\left(\dot{{\beta }_{d}}-\dot{\beta }+\dot{{\gamma }_{d}}\right){I}_{z}+D\right]}{{C}_{Y}+{K}_{Y}}+\frac{{K}_{Y}\left[\left[\dot{{\beta }_{d}}-\dot{\beta }+\dot{{\gamma }_{d}}+{C}_{Y}\left(\beta -{\beta }_{d}-{\gamma }_{d}+A{V}_{x}\right)\right]{I}_{z}+D\right]}{{C}_{Y}+{K}_{Y}},$$

$$\mathrm{D}=\frac{{d}_{r}\left({F}_{xrl}-{F}_{xrr}\right)}{2}+\frac{\left({d}_{f}\left(\mathrm{cos}\delta \left({F}_{xfl}-{F}_{xfr}\right)\right)-sin\delta ({F}_{yfl}-{F}_{yfr})\right)}{2}-{L}_{f}\left(cos\delta \left({F}_{yfl}+{F}_{yfr}\right)+sin\delta \left({F}_{xfl}+{F}_{xfr}\right)\right)+{a}_{y}\left({L}_{f}{m}_{f-}{L}_{r}{m}_{r}\right)+{L}_{r}\left({F}_{yfl}+{F}_{yrr}\right).$$

### ASC control law design

Design sliding mode:

55 $${s}_{A}=\dot{{e}_{A}}+{c}_{A}{e}_{A},$$

where ${c}_{A}$ is an adjustable parameter and ${c}_{A}>$ 0; ${e}_{A}$ is the roll angle tracking error.

Similarly, the saturation function $sat\left({s}_{s}\right)$ is used to replace the symbol function $sgn\left({s}_{s}\right)$^28^, and the exponential reaching rate is as follows:

56 $$\dot{{s}_{A}}=-{\varepsilon }_{A}\cdot sat\left({s}_{A} \right)-{k}_{A}{s}_{A},$$

where ${\varepsilon }_{A}$ and ${k}_{A}$ are adjustable parameters.

Combining Eqs. (35), (55), and (56), the expected roll control torque $\Delta {M}_{\mathcal{X}}$ sliding mode control law is calculated as follows:

57 $$\Delta {M}_{\mathcal{X}}=-{I}_{x} \left[{\varepsilon }_{A} sat-{\ddot{\varnothing }}_{d}+{c}_{A}\left(\dot{\varnothing }-{\ddot{\varnothing }}_{d}\right)+{k}_{A}\left(\dot{\varnothing }-{\ddot{\varnothing }}_{d}\right)+{c}_{A}\left(\varnothing -{\varnothing }_{d}\right)\right]+\frac{{C}_{\varnothing }\dot{\varnothing }+{I}_{XZ}\dot{\gamma }+\varnothing \left({K}_{\varnothing }-g{h}_{b}{m}_{b}\right)+{a}_{y}{h}_{b}{m}_{b}}{{I}_{x}}.$$

Just to be clear, the average front wheel angle, expected longitudinal force, expected yaw control torque, and expected roll control torque control law obtained above need to be further transformed into steering wheel angle, wheel driving torque, wheel braking torque and quarter suspension actuation force to achieve control. The transformation principles are as follows: according to the average angle of the front wheel calculated by PFC, the steering wheel angle is obtained by the relationship curve between the average angle of the front wheel and the steering wheel angle; according to the expected longitudinal force calculated by STC, the front and rear wheel torques are allocated according to the axial load ratio distribution, and then the left and right wheel torques are allocated according to the average distribution, and the driving or braking torques are output; according to the additional yaw torque calculated by YSC, the torque distribution method based on wheel load is adopted to allocate the wheel torque^29^; according to the roll control torque calculated by ASC, the front and rear suspensions are distributed according to the axial load ratio, and then the left and right suspensions are distributed according to the average distribution. Because the above distribution principle is simple or there are corresponding references, it is no longer redundant.

## Demonstrative example

In the intelligent network environment, the key technologies of automobile are more abundant, including environmental perception technology, intelligent decision-making technology, control execution technology and system design technology. The research on these key technologies has brought new solutions to vehicle dynamic performance control. DDAVs carry a wealth of sensing units, high-precision maps, full wheel drive system and active suspension system. According to the existing research, the road adhesion coefficient can be predicted by identifying the front road with lidar and camera^35^. Real-time path planning^36^ and vehicle speed planning^37^ can be realized through the planning and decision-making system and high-precision maps of the intelligent vehicles. In practical application, the road adhesion coefficient, tracking path and target speed can be obtained in advance as the input of the proposed control method. Moreover, the advanced vehicle control unit of intelligent vehicle provides a computational guarantee for the control method proposed in this paper. The control system proposed in this paper mainly controls the steering system, driving system, braking system, and active suspension system of DDAVs. To verify the feasibility of the proposed control method through simulation, it is assumed that the adhesion coefficient of the front road, the tracking path, and the target vehicle speed have been obtained in advance.

**Figure 3:**
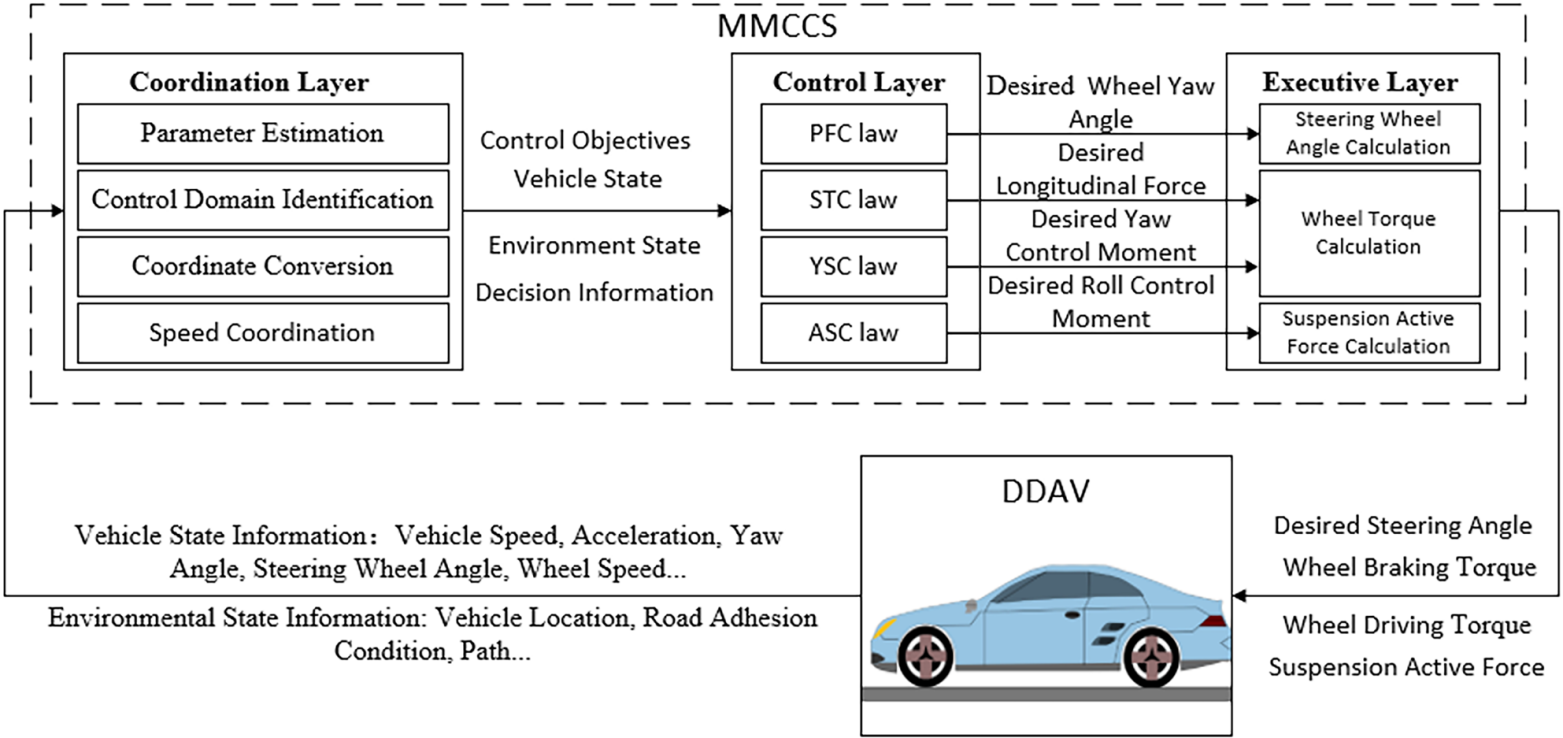
Architecture of multidirectional motion coupling control system.

To verify the effectiveness and superiority of MMCCS designed based on the proposed extreme speed estimation method and motion control law design method, an example DDAV model is used for simulation verification. MMCCS architecture adopts conventional hierarchical control architecture, which is divided into coordination layer, control layer, and execution layer, as shown in Fig. 3. The coordination layer mainly completes four tasks: the parameter estimation mainly completes the state parameter estimation of the control layer requirements such as tire force, sideslip angle, and wheel slip rate; control domain identification mainly completes dynamic boundary calculation, to identify DDAV driving stability; coordinate transformation mainly completes GPS information acquisition and transformation into geodetic coordinate information; speed coordination mainly completes the identification of stable driving extreme speed under driving conditions, and coordinates with the driving speed given by the automatic driving decision layer, and finally plans the safe driving speed. The control layer mainly calculates the expected vehicle longitudinal force, wheel angle, yaw control torque, and roll control torque based on the multidirectional motion coupling control method. The executive layer further calculates the driving torque of each wheel, braking torque of each wheel, steering angle, and suspension force according to the calculation results of the control layer. The main parameters of the example DDAV model are shown in Table 1. The vehicle dynamics simulation model is established in LMS Imagine. Lab Amesim. MMCCS model was established in Matlab/Simulink. The vehicle dynamics simulation model and control system model constitute the DDAVs multidirectional motion control simulation model.

**Table 1:** Main DDAV parameters.

Symbol	Description	Value
m	Vehicle total mass	1430 (kg)
a	Distance from center of gravity to front axle	1.056 (m)
b	Distance from center of gravity to rear axle	1.344 (m)
${d}_{f}$	Distance between front left and right wheels	1.45 (m)
${d}_{r}$	Distance between rear left and right wheels	1.45 (m)
R	Tire rolling radius	0.29 (m)
${h}_{g}$	Height of vehicle center of gravity	0.675 (m)
${I}_{z}$	Yaw moment of inertia of vehicle	1300 (kg m^2^)
${J}_{w}$	Moment of inertia of wheel	0.85 (kg m^3^)
${C}_{f}$	Equivalent nominal front tire cornering stiffness	55,634 (N/rad)
${C}_{r}$	Equivalent nominal rear tire cornering stiffness	50,764 (N/rad)

**Figure 4:**
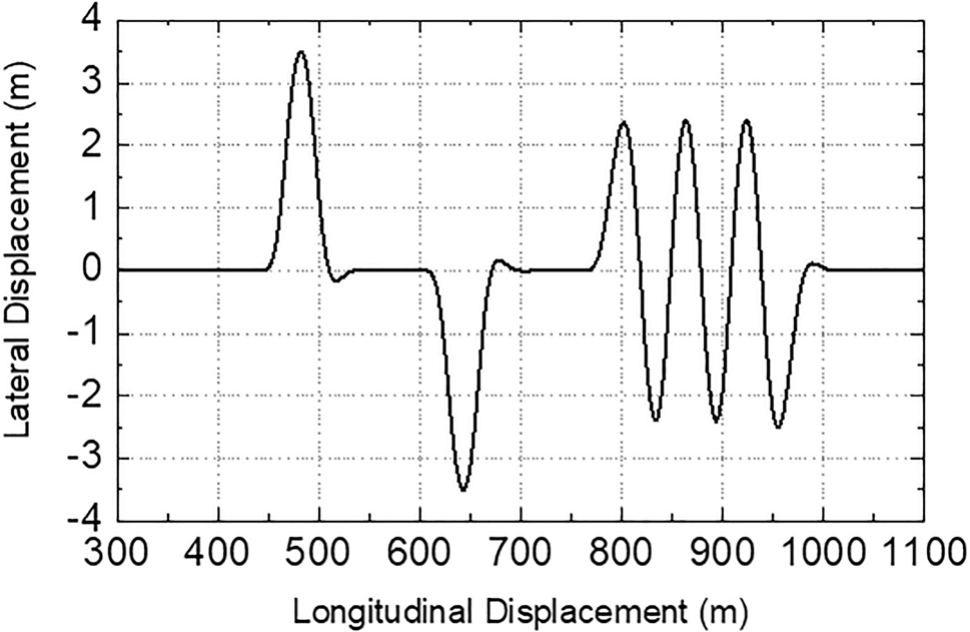
Tracking path.

The double-line-shift and serpentine driving conditions are used for simulation verification, and the designed path is shown in Fig. 4. In the simulation process, the simulation time step is 0.01 s, and three different roads are selected for simulation analysis. The selected three kinds of roads are: the dry road, the corresponding road adhesion coefficient is 0.8; the wet road, the corresponding road adhesion coefficient is 0.5; the ice road, the corresponding road adhesion coefficient is 0.15.

### Validity verification

To verify the effectiveness of the proposed extreme speed estimation method and motion control law design method, MMCCS composed of PFC, STC, YSC, and ASC is used for simulation, which shows that MMCCS can exert the control potential brought by multi-degree-of-freedom controllability of DDAVs, and meet the requirements of trajectory tracking control accuracy and driving stability control of DDAVs. To facilitate the description, the DDAVs motion control system with only PFC and STC is called Simple Control System (SCS).

**Figure 5:**
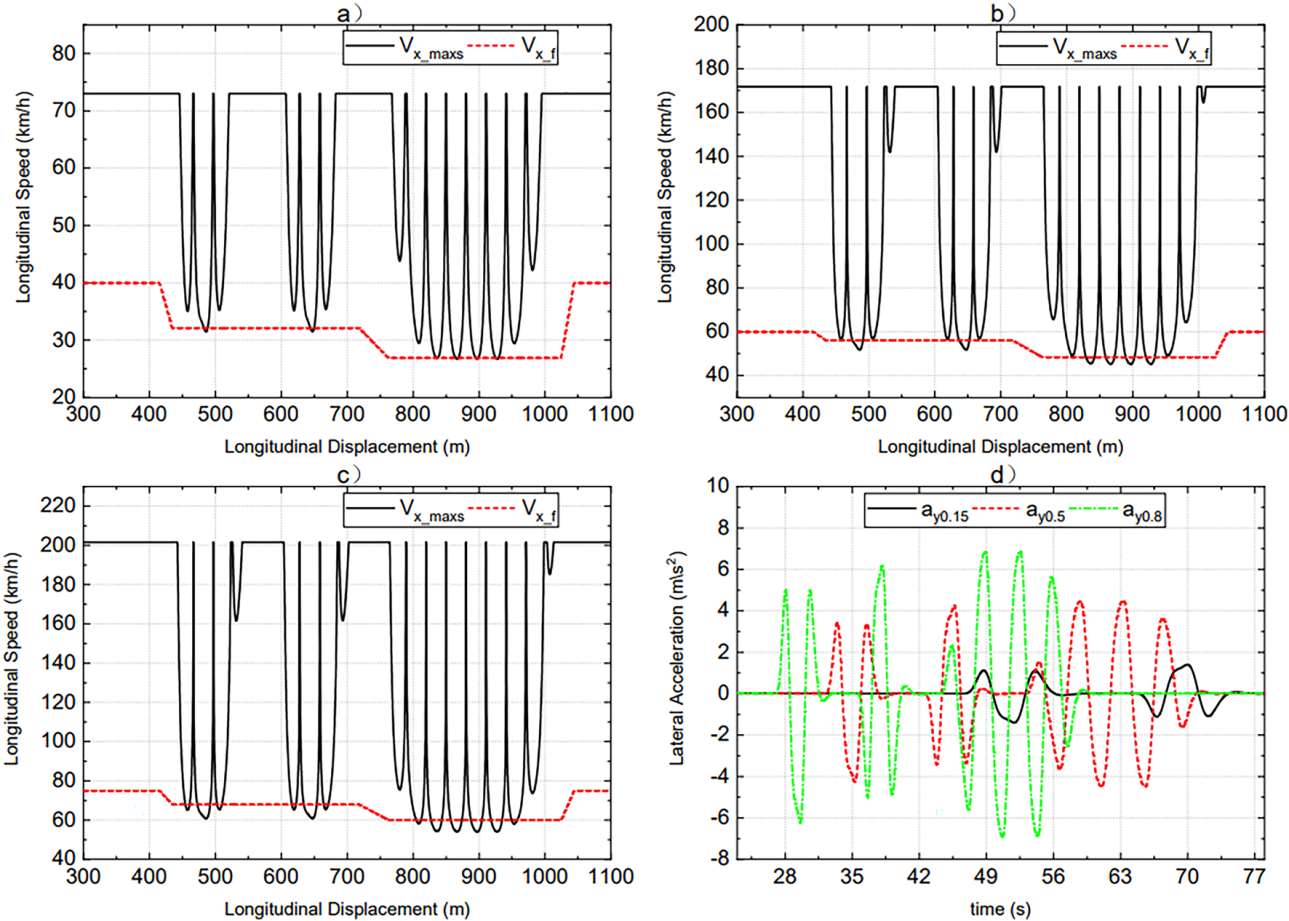
(**a**–**c**) are the speed preview results with road adhesion coefficients of 0.15, 0.5, and 0.8 under the given path, (**d**) is the lateral acceleration of DDAVs under these three road adhesion coefficients when SCS is used.

**Figure 6:**
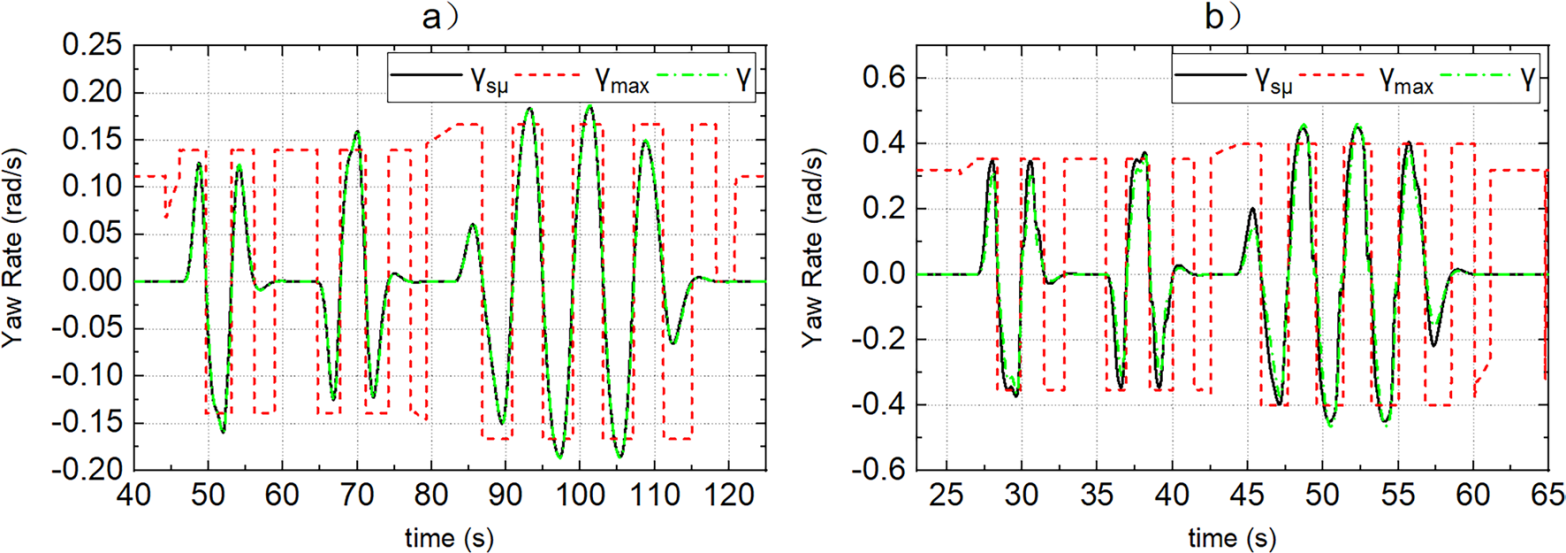
(**a**, **b**) Represent the response of yaw rate of dynamic boundary and actual yaw rate when the road adhesion coefficients are 0.15 and 0.8, respectively.

**Figure 7:**
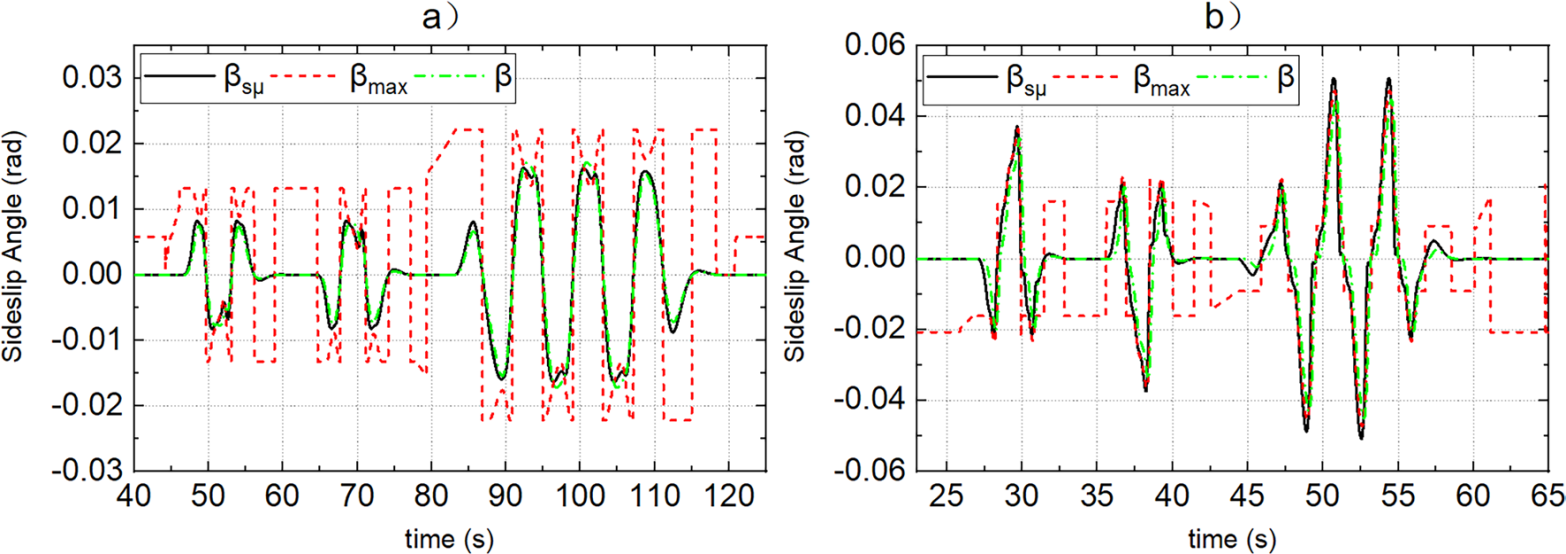
(**a**, **b**) Represent the response of sideslip angle of dynamic boundary and actual sideslip angle when the road adhesion coefficients are 0.15 and 0.8, respectively.

**Figure 8:**
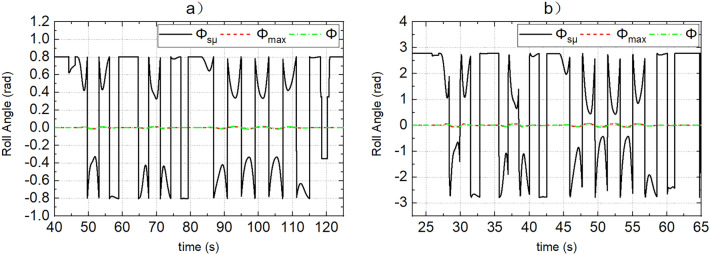
(**a**, **b**) Represent the response of roll angle of dynamic boundary and actual roll angle when the road adhesion coefficients are 0.15 and 0.8, respectively.

DDAVs travel along the path shown in Fig. 4. When the road adhesion coefficient is different, the safe extreme speed ${V}_{x\_maxs}$ and the final expected speed ${V}_{x\_f}$ are shown in Fig. 5a–c The starting and ending values of ${V}_{x\_f}$ in Fig. 5a–c are determined by the expected speed ${V}_{x\_p}$ of the decision layer. When SCS is used, DDAVs travel at the final expected speed under the set conditions, the lateral acceleration response is shown in Fig. 5d, and the dynamic boundary is shown in Figs. 6, 7 and 8. By analyzing the data in the figures, the following conclusions can be drawn:

1. Figures 5c, 6, 7 and 8 show that when DDAVs are driven by ${V}_{x\_f}$, the dynamic response exceeds the unstable boundary under extreme conditions, and the lateral acceleration has reached a large value, but is still within the controllable range, which proves that the vehicle speed has almost reached the maximum allowable speed for safe operation. It indicates that the final expected speed calculated by the speed coordination strategy reaches the design goal of DDAVs stable extreme speed.
2. Figure 5a–c shows that the extreme speed estimation algorithm can estimate the safety extreme speed under continuous change conditions according to different road adhesion coefficients and different road curvatures. The extreme speed estimation algorithm not only determines the final expected speed, but also lays a foundation for the control potential of MMCCS. And as far as possible to meet the decision layer expected speed requirements, help to improve the efficiency of automobile transportation. From the design method of the speed coordination strategy, it can be seen that this strategy mainly relies on the data mapping table in operation, and can preview the speed after the path is determined, which is conducive to improving the real-time control of MMCCS.

The above conclusions show that the proposed extreme speed estimation method based on the dynamic boundary can identify the maximum stable speed of DDAVs under extreme conditions, and can plan the safe speed of the vehicle under the premise of meeting the speed demand of the decision layer.

**Figure 9:**
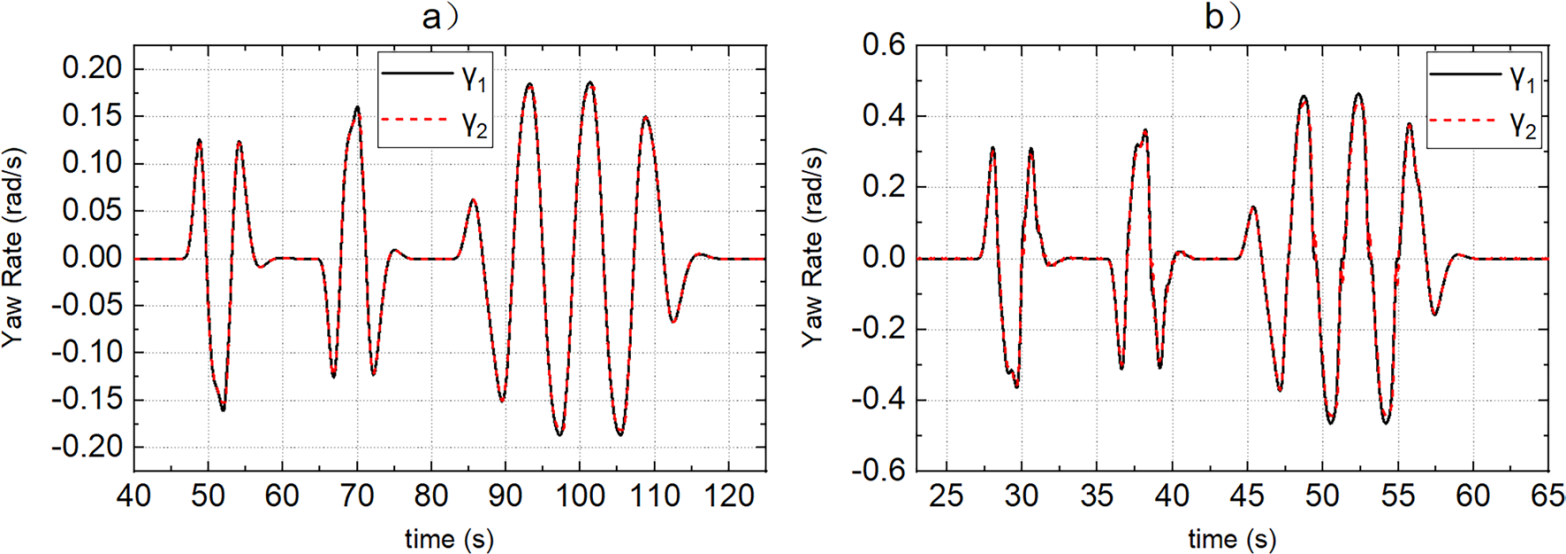
(**a**,**b**) Show the actual yaw rate when the road adhesion coefficients are 0.15 and 0.8, respectively. Curve 1 represents the SCS effect, and curve 2 represents the MMCCS effect.

**Figure 10:**
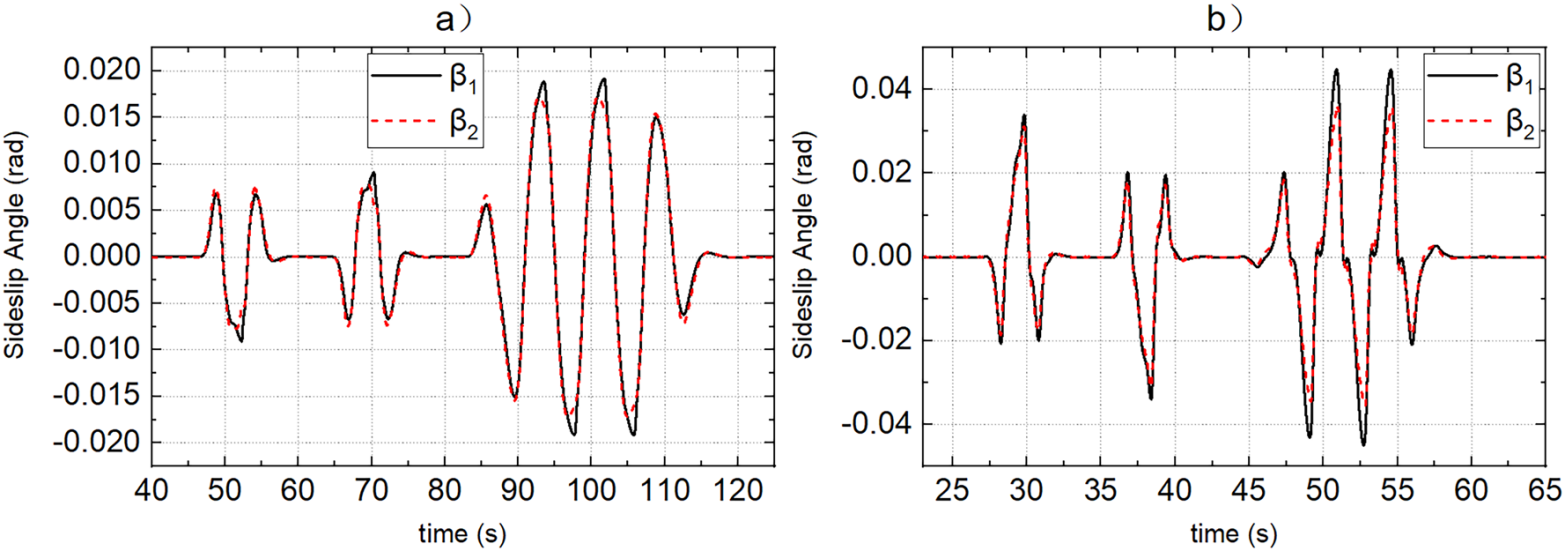
(**a**,**b**) Show the actual sideslip angle when the road adhesion coefficients are 0.15 and 0.8, respectively. Curve 1 represents the SCS effect, and curve 2 represents the MMCCS effect.

**Figure 11:**
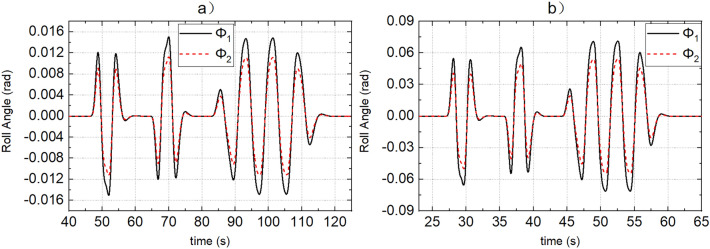
(**a**,**b**) Show the actual roll angle when the road adhesion coefficients are 0.15 and 0.8, respectively. Curve 1 represents the SCS effect, and curve 2 represents the MMCCS effect.

**Figure 12:**
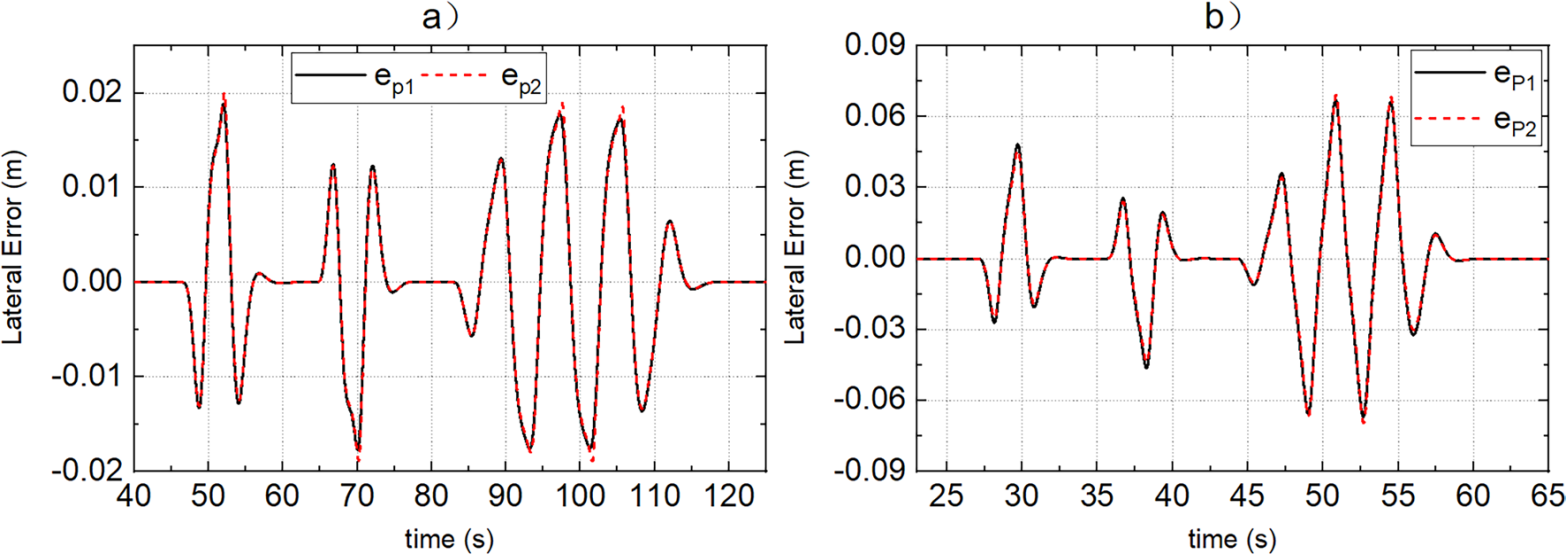
(**a**,**b**) Show the path following error when the road adhesion coefficients are 0.15 and 0.8, respectively. Curve 1 represents the SCS effect, and curve 2 represents the MMCCS effect.

**Figure 13:**
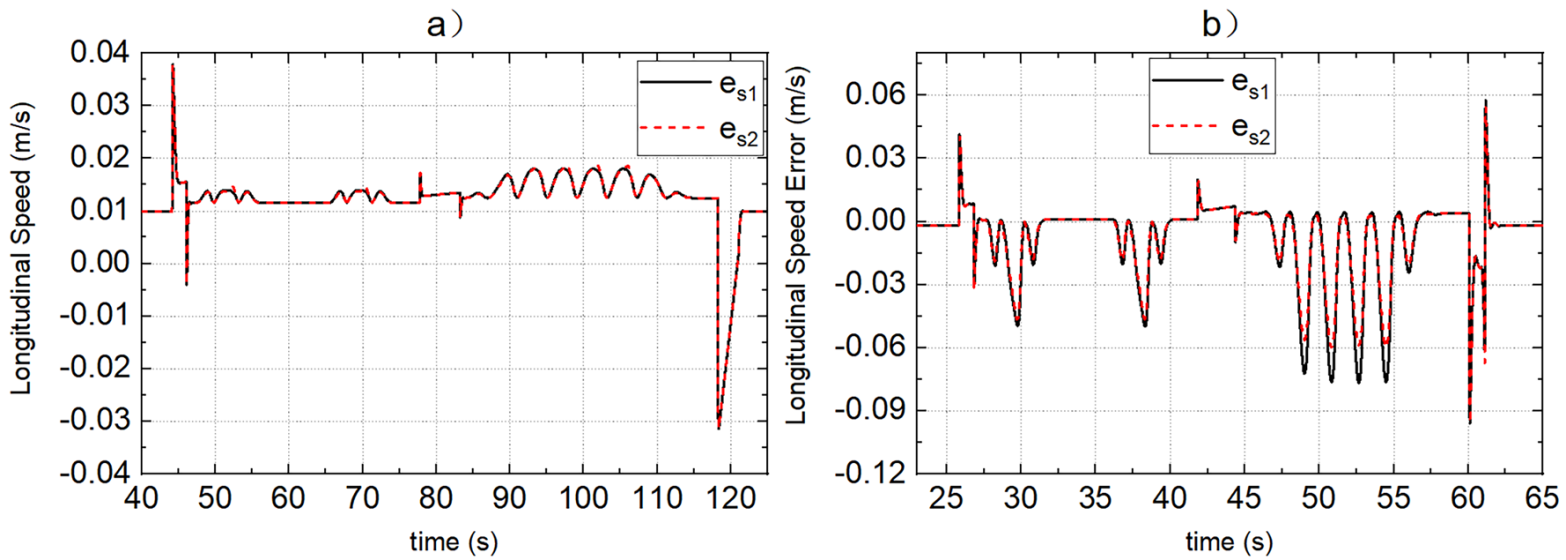
(**a**,**b**) Show the speed tracking error when the road adhesion coefficients are 0.15 and 0.8, respectively. Curve 1 represents the SCS effect, and curve 2 represents the MMCCS effect.

SCS and MMCCS are used for simulation respectively, and the simulation results of yaw rate, sideslip angle, roll angle, path tracking error, and vehicle speed tracking error are shown in Figs. 9, 10, 11, 12 and 13. Due to the limited space, Figs. 9, 10, 11, 12 and 13 shows only the simulation results of road adhesion coefficients of 0.8 and 0.15. The simulation results of MMCCS and SCS are sorted out to form DDAVs simulation results summary Table 2. The optimization ratios in Table 2 are calculated relative to the SCS control results, and are calculated according to the larger absolute values of the upper and lower boundaries of the parameter variation range. For example, when the road adhesion coefficient is 0.8, the calculation process of ${a}_{\mathcal{Y}}$ optimization ratio is as follows: because abs(− 6.906) < abs(6.907), and abs(− 6.879) > abs(6.872), so the optimization ratio is [abs(6.907) − abs(− 6.879)]/abs(6.907)$\approx$ 0.4%. By analyzing the data in Figs. 9, 10, 11, 12 and 13 and Table 2, the following conclusions are drawn:

1. SCS and MMCCS can achieve multidirectional motion control and have good control accuracy under different road adhesion coefficients and road curvature conditions. Longitudinal speed tracking error is less than 0.1 m/s; the path tracking error reaches centimeter level.
2. It can be seen from Table 2 that after MMCCS is adopted, the path tracking performance decreases, but it is still in an acceptable range, and the driving stability of the vehicle is significantly improved. Because the driving condition of DDAVs studied in this paper is the extreme condition, it is worth losing a little path tracking performance and improving driving stability. Compared with SCS, MMCCS adopts YSC and ASC, and yaw rate, sideslip angle, and roll angle decrease, so the yaw stability and roll stability of DDAVs are improved. At the same time, the lateral acceleration is reduced, which is conducive to improving the lateral stability of the vehicle. Moreover, the longitudinal speed tracking error is almost unchanged when SCS and MMCCS are used, which indicates that MMCCS can improve the stability of DDAVs without reducing the original speed tracking control accuracy.

**Table 2:** Summary of DDAVs double-line-shift and serpentine driving simulation results.

Strategy		SCS	MMCCS	Optimization ratio
$\mu$	Parameters	Range	Range	
0.8	β	[− 0.045 rad, 0.045 rad]	[− 0.036 rad, 0.036 rad]	26.70%
	γ	[− 0.465 rad/s, 0.465 rad/s]	[− 0.444 rad/s, 0.443 rad/s]	4.50%
	${e}_{P}$	[− 0.067 m, 0.067 m]	[− 0.069 m, 0.069 m]	− 2.99%
	$\phi$	[− 0.071 rad, 0.071 rad]	[− 0.054 rad, 0.054 rad]	23.90%
	${a}_{\mathcal{Y}}$	[− 6.906 m/s2, 6.907 m/s2]	[− 6.879 m/s2, 6.872 m/s2]	0.4%
	$\delta$	[− 113.855 deg, 114.036 deg]	[− 133.131 deg, 135.203 deg]	− 18.56%
	${e}_{s}$	[− 0.096 m/s, 0.058 m/s]	[− 0.096 m/s, 0.054 m/s]	0.00%
0.5	β	[− 0.015 rad, 0.015 rad]	[− 0.011 rad, 0.011 rad]	26.70%
	γ	[− 0.350 rad/s, 0.350 rad/s]	[− 0.336 rad/s, 0.335 rad/s]	4.00%
	${e}_{P}$	[− 0.041 m, − 0.041 m]	[− 0.045 m, 0.045 m]	− 9.80%
	$\phi$	[− 0.048 rad, 0.048 rad]	[− 0.036 rad, 0.036 rad]	25.00%
	${a}_{\mathcal{Y}}$	[− 4.501 m/s2, 4.502 m/s2]	[− 4.475 m/s2, 4.470 m/s2]	0.60%
	$\delta$	[− 86.099 deg, 86.152 deg]	[− 106.367 deg, 107.635 deg]	− 24.94%
	${e}_{s}$	[− 0.054 m/s, 0.067 m/s]	[− 0.054 m/s, 0.066 m/s]	1.49%
0.15	β	[− 0.019 rad, 0.019 rad]	[− 0.017 rad, 0.017 rad]	11.80%
	γ	[− 0.187 rad/s, 0.187 rad/s]	[− 0.181 rad/s, 0. 181 rad/s]	3.20%
	${e}_{P}$	[− 0.018 ms, 0.019 m]	[− 0.019 m, 0.020 m]	− 5.30%
	$\phi$	[− 0.015 rad, 0.015 rad]	[− 0.011 rad, 0.011 rad]	26.70%
	${a}_{\mathcal{Y}}$	[− 1.403 m/s2, 1.402 m/s2]	[− 1.383 m/s2, 1.382 m/s2]	1.40%
	$\delta$	[− 57.147 deg, 57.153 deg]	[− 65.432 deg, 65.775 deg]	− 15.10%
	${e}_{s}$	[− 0.031 m/s, 0.038 m/s]	[− 0.031 m/s, 0. 038 m/s]	0.00%

The above conclusions show that the proposed multidirectional motion coupling control method based on vehicle dynamics model can give full play to the advantages of multi degrees of freedom control of DDAVs. By realizing the coupling control of longitudinal motion, lateral motion, yaw motion, and roll motion of the vehicle, the multidirectional motion response performance of the vehicle is improved, and the multidirectional motion control accuracy and driving stability of DDAVs under extreme conditions are guaranteed.

### Superiority verification

The commonly used methods for the multidirectional motion control of DDAVs in the existing research are: speed tracking control and path tracking control based on 2-DOF vehicle model and model prediction algorithm; yaw stability control based on 7-DOF vehicle model and sliding mode algorithm; and roll stability control based on the half-vehicle model and sliding mode algorithm. To facilitate the description, the DDAVs multidirectional motion integrated control system designed by common vehicle models and algorithms is called the Traditional Control System (TCS). To verify the superiority of the proposed extreme speed estimation method and motion control law design method, MMCCS and TCS are used for comparative simulation analysis.

The front wheel angle control law and vehicle longitudinal force control law are designed based on the 3-DOF vehicle model and model prediction algorithm in STC. These two control laws in TCS are introduced in detail in Chapters 3 and 5 in reference^38^, which are not repeated here due to the limited space.

In TCS, the yaw moment control law based on the 7-DOF vehicle model and sliding mode algorithm is designed as follows^39^:

58 $${\Delta M}_{zcT}=\frac{-\left\{{\varepsilon }_{T}\cdot sat\left({s}_{YT} \right)+{k}_{T}\left[{\xi }_{T2}\left(\beta -{\beta }_{d}\right)+{c}_{YT}{e}_{YT}-{\xi }_{T1}\left(\dot{\gamma }+\mathrm{A}\right)\right]\right\}}{\left({c}_{Y2+}{k}_{3}\right)\frac{{\xi }_{3}}{{I}_{z}}}-\frac{\left\{{\xi }_{T2}\ddot{{e}_{YT}}+{c}_{YT}\left[{\xi }_{T2}\left(\beta -{\beta }_{d}\right)-{\xi }_{T}\left(\dot{\gamma }+\mathrm{A}\right)\right]\right\}}{\left({c}_{YT+}{k}_{T}\right)\frac{{\xi }_{T1}}{{I}_{z}}}.$$

In the Eq. (58):

$$A=\frac{\frac{{d}_{r}}{2}\left({F}_{\mathcal{X}rl}-{F}_{\mathcal{X}rr}\right)+\frac{{d}_{f}}{2}\left[\left({F}_{\mathcal{X}fl}-{F}_{\mathcal{X}fr}\right)\mathit{cos}\delta -\left({F}_{\mathcal{Y}fl}-{F}_{\mathcal{Y}fr}\right)\mathit{sin}\delta \right]}{{I}_{z}}-\frac{a\left[\left({F}_{\mathcal{Y}fr}+{F}_{\mathcal{Y}fl}\right)\mathit{cos}\delta +\left({F}_{\mathcal{X}fr}+{F}_{\mathcal{X}fl}\right)\mathit{sin}\delta \right]+ b\left({F}_{\mathcal{Y}rl}+{F}_{\mathcal{Y}rr} \right)}{{I}_{z}},$$

where ${s}_{YT}$ is sliding mode; ${e}_{YT}$ is control error; ${c}_{YT}$, ${k}_{T}$, ${\varepsilon }_{T}$ are adjustable parameters; ${\xi }_{T1}$ and ${\xi }_{T2}$ are weight coefficients, ${\gamma }_{dt}$ and ${\beta }_{dt}$ are control objectives.

In TCS, the vehicle roll torque control law is designed based on the half-vehicle model and integral sliding mode algorithm as follows^40^:

59 $$\Delta {M}_{\mathcal{X}T}={I}_{x}\left[\frac{{m}_{\mathrm{b}}\left({a}_{y}+g\varnothing \right){h}_{\mathrm{b}}-{D}_{\varnothing }\dot{\varnothing }-{K}_{\varnothing }\varnothing }{{I}_{x}}+{k}_{1}\dot{\varnothing }+{k}_{2}\varnothing +{c}_{YT}(\dot{\varnothing }+\left.\left.{k}_{1}\varnothing +{k}_{2}\cdot \int \varnothing \mathrm{d}t\right)-{\varepsilon }_{YT}\cdot \mathrm{sgn}\left({s}_{YT}\right)\right]\right.,$$

where ${\varepsilon }_{YT}$ and ${c}_{YT}$ are positive constant; ${k}_{1}$ and ${k}_{2}$ are nonzero positive constant; ${s}_{YT}$ is an integral sliding surface.

The multidirectional motion control performance of DDAVs can be evaluated by the control error $e\left(t\right)$ and the control actuation $\Delta X$. To facilitate comparison, the control error and control momentum are processed as follows^13^:

Integrate the absolute value of the error in the simulation period:

60 $$IAE=\int \nolimits_{t1}^{t2}\left|e(t)\right|dt.$$

The absolute value of the error is weighted by time and integrated within the simulation time period:

61 $$ITAE=\int \nolimits_{t1}^{t2}t\left|e(t)\right|dt.$$

Integrate the absolute value of control actuation within the simulation period:

62 $$IACA=\int \nolimits_{t1}^{t2}\left|\Delta X\right|dt.$$

In Eqs. (60) and (61), $e(t)$ denotes ${e}_{P}$, ${e}_{s}$, ${e}_{\gamma }$, ${e}_{\beta }$ and ${e}_{A}$; $t1$ and $t2$ denote the time when DDAVs enter and exit the focus condition; when $e\left(t\right)={e}_{P}$, ${IAE}_{p}$ and ${ITAE}_{p}$ exist; when $e\left(t\right)={e}_{s}$, ${IAE}_{s}$ and ${ITAE}_{s}$ exist; when $e\left(t\right)={e}_{\gamma }$, ${IAE}_{\gamma }$ and ${ITAE}_{\gamma }$ exist; when $e\left(t\right)={e}_{\beta }$, ${IAE}_{\beta }$ and ${ITAE}_{\beta }$ exist; when $e\left(t\right)={e}_{A}$, ${IAE}_{A}$ and ${ITAE}_{A}$ exist. In Eq. (62), $\Delta X$ denotes $\delta$, ${F}_{xc}$, ${\Delta M}_{z}$ and $\Delta {M}_{\mathcal{X}}$; when $\Delta X=\delta$, ${IACA}_{\delta }$ exists; when $\Delta X={F}_{xc}$, ${IACA}_{Fx}$ exists; when $\Delta X={\Delta M}_{z}$, ${IACA}_{MZ}$ exists; when $\Delta X={\Delta M}_{x}$, ${IACA}_{Mx}$ exists.

**Figure 14:**
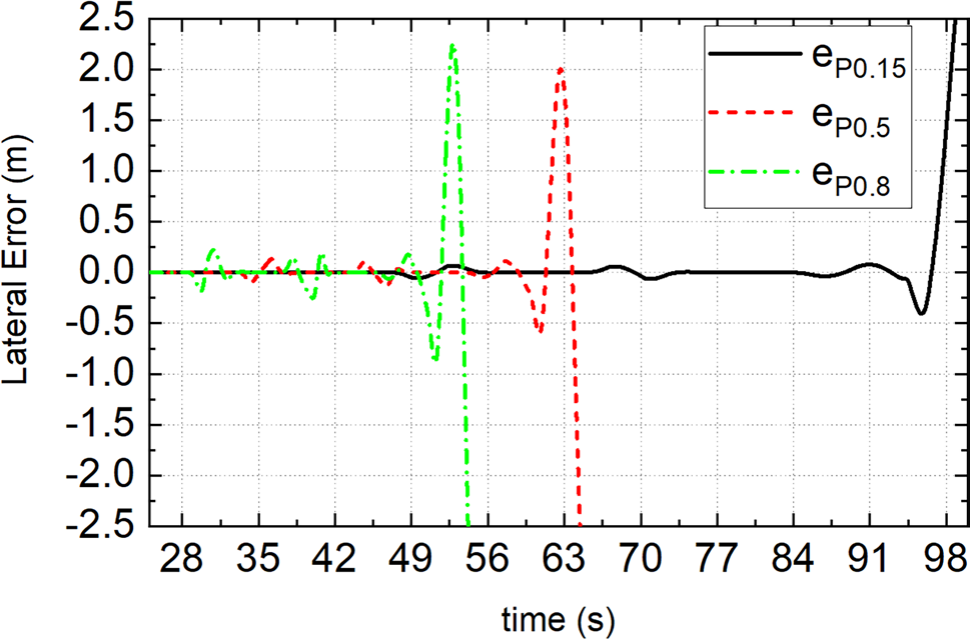
Path tracking error when TCS is adopted.

Due to the limited space, only the simulation path tracking error is shown in Fig. 14. Figure 14 shows that under given conditions, MMCCS can achieve DDAVs multidirectional motion control at extreme speed, TCS can complete double-line-shift driving control at extreme speed, and the DDAV runs out of the lane under the serpentine driving condition. It is proved that the proposed multidirectional motion coupling control method has better working condition adaptability than the traditional integrated control method, and can give full play to the multi degrees of freedom controllable advantages of DDAVs, which is more conducive to improving the driving stability of DDAVs. The evaluation results of the simulation under the double-line-shift condition are shown in Table 3. By analyzing the data in Table 3 and comparing the evaluation indexes of ${e}_{P}$ and $\delta$, it is found that the path tracking ability is MMCCS > TCS. By comparing the evaluation indexes of ${e}_{\beta }$, ${e}_{\gamma }$ and ${\Delta M}_{z}$, it can be seen that the control ability of yaw stability is MMCCS > TCS. Compared with the evaluation indexes of ${e}_{s}$ and ${F}_{xc}$, the speed tracking ability is MMCCS > TCS. Comparing the evaluation indexes of ${e}_{A}$ and ${\Delta M}_{x}$, it can be seen that the roll stability control ability is MMCCS > TCS. Overall, MMCCS has better DDAVs multidirectional motion control accuracy and driving stability control effect than TCS.

**Table 3:** Evaluation index statistics of double-line-shift driving simulation.

Strategy		SCS			MMCCS		
$\mu$	Parameters	IAE	ITAE	IACA	IAE	ITAE	IACA
0.8	${e}_{P}$	0.892	31.588	–	0.153	5.157	–
	${e}_{s}$	1.093	35.165	–	0.129	4.230	–
	${e}_{\gamma }$	0.323	11.390	–	0.317	10.690	–
	${e}_{\beta }$	0.056	2.014	–	0.044	1.473	–
	${e}_{A}$	0.119	4.219	–	0.095	3.160	–
	$\delta$	–	–	0.408	–	–	0.358
	${F}_{xc}$	–	–	7504.000	–	–	6948.000
	${\Delta M}_{z}$	–	–	9505.920	–	–	4710.583
	${\Delta M}_{x}$	–	–	8294.000	–	–	6952.000
0.5	${e}_{P}$	0.532	21.766	–	0.147	5.896	–
	${e}_{s}$	0.627	27.739	–	0.092	3.686	–
	${e}_{\gamma }$	0.202	8.215	–	0.213	8.578	–
	${e}_{\beta }$	0.200	0.821	–	0.0183	0.741	–
	${e}_{A}$	0.057	2.319	–	0.047	1.858	–
	$\delta$	–	–	0.384	–	–	0.369
	${F}_{xc}$	–	–	4408.000	–	–	4226.000
	${\Delta M}_{z}$	–	–	4016.812	–	–	2988.667
	${\Delta M}_{x}$	–	–	6348.000	–	–	5656.000
0.15	${e}_{P}$	0.567	34.341	–	0.162	9.757	–
	${e}_{s}$	0.250	14.406	–	0.391	24.275	–
	${e}_{\gamma }$	0.105	6.485	–	0.106	6.550	–
	${e}_{\beta }$	0.023	1.412	–	0.023	1.408	–
	${e}_{A}$	0.007	0.446	–	0.005	0.314	–
	$\delta$	–	–	0.430	–	–	0.441
	${F}_{xc}$	–	–	1836.000	–	–	1658.000
	${\Delta M}_{z}$	–	–	1441.178	–	–	1366.778
	${\Delta M}_{x}$	–	–	3291.000	–	–	3169.000

## Conclusion

In this paper, the extreme speed estimation method based on dynamic boundary and the multidirectional motion coupling control law design method based on multi degrees of freedom vehicle dynamic model are proposed. The simulation results show that MMCCS designed based on the proposed methods can identify and plan the stable driving extreme speed of the vehicle under different working conditions, and can realize the multidirectional motion coupling control of DDAV, which ensures that DDAV has good motion control accuracy and driving stability under different road adhesion coefficients and different curvatures of the road. In future work, the dynamic boundary will be further improved to introduce more stability evaluation factors into the extreme speed estimation method, so that the designed extreme speed can consider the stability of DDAV more comprehensively. At the same time, considering other controllable degrees of freedom of DDAV, a more comprehensive multidirectional motion coupling control law design method based on multi degrees of freedom vehicle dynamic model will be studied.

## Data Availability

The datasets generated and/or analyzed during the current study are available from the corresponding author on reasonable request.
